# Single and combined effect of retinoic acid and rapamycin modulate the generation, activity and homing potential of induced human regulatory T cells

**DOI:** 10.1371/journal.pone.0182009

**Published:** 2017-07-26

**Authors:** Enzo Candia, Paz Reyes, Camila Covian, Francisco Rodriguez, Nicolas Wainstein, Jorge Morales, Claudio Mosso, Mario Rosemblatt, Juan Alberto Fierro

**Affiliations:** 1 Centro de Trasplantes, Clinica Las Condes, Santiago, Chile; 2 Fundación Ciencia & Vida, Santiago, Chile; 3 Facultad de Ciencias Biologicas, Universidad Andres Bello, Santiago, Chile; 4 Departamento de Biología, Facultad de Ciencias, Universidad de Chile, Santiago, Chile; Beth Israel Deaconess Medical Center, UNITED STATES

## Abstract

Adoptive transfer of CD4+CD25+FOXP3+ regulatory T cells (Treg cells) has been successfully utilized to treat graft versus host disease and represents a promising strategy for the treatment of autoimmune diseases and transplant rejection. The aim of this study was to evaluate the effects of all-trans retinoic acid (atRA) and rapamycin (RAPA) on the number, phenotype, homing markers expression, DNA methylation, and function of induced human Treg cells in short-term cultures. Naive T cells were polyclonally stimulated and cultured for five days in the presence of different combinations of IL-2, TGF-β1, atRA and RAPA. The resulting cells were characterized by the expression of FOXP3, activation, surface and homing markers. Methylation of the Conserved Non-coding Sequence 2 was also evaluated. Functional comparison of the different culture conditions was performed by suppression assays in vitro. Culturing naive human T cells with IL-2/TGFβ1 resulted in the generation of 54.2% of Treg cells (CD4+CD25+FOXP3+) whereas the addition of 100 nM atRA increased the yield of Treg cells to 66% (p = 0.0088). The addition of RAPA did not increase the number of Treg cells in any of these settings. Treg cells generated in the presence of atRA had an increased expression of the β7 integrin to nearly 100% of the generated Treg cells, while RAPA treated cells showed enhanced expression of CXCR4. The differential expression of homing molecules highlights the possibility of inducing Treg cells with differential organ-specific homing properties. Neither atRA nor RAPA had an effect on the highly methylated CNS2 sites, supporting reports that their contribution to the lineage stability of Treg cells is not mediated by methylation changes in this locus. Treg cells generated in the presence of RAPA show the most potent suppression effect on the proliferation of effector cells.

## Introduction

The discovery, isolation, and generation of CD4+CD25+FOXP3+ regulatory T cells (Treg cells) represent a remarkable breakthrough in modern immunology[1,2]. Regulatory T cells (Treg cells) are crucial players for the maintenance of peripheral tolerance, controlling activation and expansion of autoreactive cells, therefore performing a vital contribution to the control of autoimmunity [3]. Since it is possible to expand Treg cells *ex vivo*, an increasing number of preclinical and clinical trials have evaluated the administration of Treg cells in autoimmune diseases such as type 1 diabetes [4], systemic lupus erythematosus [5], rheumatoid arthritis [3] and Crohn’s disease [6,7]. Furthermore, in the context of organ and bone marrow transplantation, Treg cells contribute critically to the acceptance of allogeneic implants [8]. In this regard, the adoptive transfer of Treg cells in animal models of transplantation has shown to improve graft survival [9,10]. In humans, the administration of Treg cells in the setting of hematopoietic cell transplantation resulted in a reduced incidence and severity of graft-versus-host disease [11–13]. Thus, the manufacture and use of Treg cells for the advance of solid organ transplantation is being evaluated in a large cooperative project, the “ONE Study” [14,15].

Several limitations must be overcome to convert human Treg cell therapy into an efficient and safe procedure [16]. These include; the production of a high, clinically useful number of Treg cells while maintaining a stable lineage with very low, ideally absent, level of contaminating cells, the suitability of providing Treg cells with specific homing receptors depending on the clinical context, and providing Treg cells with antigen specificity. Additionally, the production of Treg cells should proceed in adherence to “good manufacturing practices,” which also relates to regulatory issues.

Currently, two main strategies to harvest Treg cells *ex vivo* appear suitable for clinical applications: the expansion of differentiated Treg cells, and the induction of Treg cells starting from naïve T cells (Tn cells). To date, it is not clear which would be the most effective and safe strategy, since both paths have potential benefits and shortcomings. Because Treg cells are scarce in peripheral blood, the generation of Treg cells from Tn cells offers the advantage of initiating the expansion from a larger cell number. Additionally, the differentiation process of Tn cells may facilitate the manipulation of cells to pursue the expression of defined homing receptors depending on the desired clinical application. Moreover, it has been reported that induced Treg cells, under certain conditions, may offer greater stability and functionality in comparison with natural Treg cells, [17]. Our study represents an effort to define the optimal conditions for the generation and expansion of Treg cells from Tn cells.

TGF-β1 and IL-2 have been shown to act as key cytokines on the differentiation of naïve T lymphocytes into Treg cells [18]. However, Treg cells generated *ex vivo* with TGF-β1 and IL-2 result in a population containing different subsets, and additionally, the induced Treg cells appear more prone to lose FOXP3 (Forkhead Box P3) expression [19]. All-Trans-Retinoic Acid (atRA), a metabolic product of retinol, in conjunction with TGF-β1 and IL-2 has been shown in several studies to improve the differentiation of Tn cells into Treg cells [20–26]. This effect is reflected in an increased number of Treg cells as well as on increased expression and stability of the main gene *FOXP3* (reviewed by Brown and Noelle [27] and Bono et al. [28]). The gene *FOXP3* maps to the *p* arm of the X chromosome, codifying the protein FOXP3, which is a transcription factor capable of modifying the expression of over 600 genes involved in the Treg cell-specific signature, therefore functioning as a “master regulator” of Treg cells [29–32].

It has also been reported that atRA blocks the production of pro-inflammatory cytokines by antigen-presenting cells (APC) [25,33], which may contribute to the improved induction of Treg cells. We aimed to establish the optimal culture conditions and the effect of different atRA concentrations on the phenotypic and functional features of atRA induced Treg cells.

Rapamycin (RAPA), an mTOR inhibitor widely used in clinical medicine, has also been shown to promote Treg cell induction, expansion and function, simultaneously inhibiting Th17 differentiation [34–36], hence presenting a possibly synergistic effect with atRA. The combined effect of both reagents has been tested in mice [37], in differentiated human Treg cells [38], and recently also in human TGF- β1 and IL-2 induced Treg cells [39]. The present study aimed to compare the effect of atRA and RAPA separately, as well as in different combinations on the number, phenotype and suppressive function of the induced Treg cells.

Additionally, the phenotypic features of human Treg cells generated under different conditions, including the expression of activation and homing molecules, need further elucidation. atRA is a major molecule involved in the induction of the intestinal homing molecules α4β77 and CCR9 [40]. It has been reported that RAPA is also able to modulate homing molecules in animal models [37], and in expanded human Treg cells [38,41], whereas its effects on induced human Treg cells require elucidation. Therefore, we were interested in determining whether atRA or RAPA as single agents or in combination could modulate the expression of specific homing molecules in the setting of induced human Treg cells generated *ex vivo*.

To exert their function, Treg cells require the concomitant expression of various transcription factors, which include the expression of the critical factor *FOXP3* [42]. Regulation of the expression of transcription factors occurs at the epigenetic level, whereas modifications of chromatin interactions, histone modifications, non-coding RNAs, nucleosome positioning, conformational changes of the chromosomes and DNA methylation play a crucial role allowing or repressing the expression of genes [43]. While histone modifications are relatively transient, DNA methylation is stable and heritable through cell divisions. At the DNA level, three evolutionarily conserved non-coding sequences (CNS) within the *FOXP3* locus contribute to regulating the induction, differentiation and heritable maintenance of the expression of *FOXP3* [44]. Consequently, the differentiation, composition, and stability of Treg cells are modulated by these CNS. Upstream of exon 1, the conserved non-coding sequence 2 (CNS2), one of the Treg cell-specific demethylated regions, is crucial for the heritable expression of FOXP3 [45]. Stable and fully functional Treg cells show the unique feature of a demethylated CNS2, and to achieve a stable heritable expression of *FOXP3* a Treg cell-specific demethylated pattern is required [46]. Indeed, *in vitro* induced Treg cells express FOXP3, although this expression may not be stable in the progeny. Thymic Treg cells hold a demethylated pattern in CNS2, and it has been shown that the acquisition of this pattern in the thymus is crucial for their stability [47]. Although other Treg cell-specific demethylated regions (TSDRs) comprising several other genes have been identified [48], the stable expression of the critical gene FOXP3 depends on the demethylated status of the CNS2. Therefore, to generate stable Treg cells *ex vivo*, its highly-methylated nature and its associated functional instability constitute challenges that need to be addressed and solved.

Nuclear retinoid receptors act as transcription factors for the expression of certain genes, usually facilitating the binding of transcriptional activators and chromatin remodeling [49]. It has also been shown that FOXP3 expression is modulated by retinoic acid [21]. In this regard, Lu *et al*. defined the mechanisms by which retinoic acid promotes the expression of FOXP3 and maintenance of the regulatory function in mice [50]. Also, Schmidt *et al*. [39] recently showed that atRA alone or with RAPA did not diminish the methylation of the Treg-specific demethylated region (TSDR). We aimed to explore whether different combinations of atRA and RAPA were able to modify the methylation status of specific CpG sites located in the CNS2.

Finally, we aimed to compare the suppressive function of the Treg cells induced *in vitro* with atRA or RAPA alone or with both agents combined, in addition to IL-2 and TGF- β1.

In summary, the main objectives of this study were to assess the effects of atRA and RAPA as single agents or combined, on the number, phenotype, and expression of activation and homing molecules. We also determined their effect on the methylation status of the CNS2, and on the suppressive function of induced polyclonal human Treg cells *in vitro*.

The results show that 100nM atRA enhanced the generation of Treg cells, increasing the number and percentage of FOXP3 expressing cells. The addition of RAPA did not contribute to increasing the number of Treg cells in this short-term experimental setting. Cultures in the presence of atRA increased the expression of the β7 integrin chain to nearly 100% on the generated Treg cells while RAPA-treated cells showed enhanced expression of CXCR4. The differential expression of homing molecules highlights the possibility of inducing Treg cells with differential organ-specific homing properties.

atRA, as well as RAPA, had no effect on the highly methylated CNS2 site, whereas the CpG sites analyzed showed all a similar behavior, suggesting that their reported contribution to the lineage stability of Treg cells is not mediated by methylation changes in this locus.

Comparison of the suppressive capacity of Treg cells obtained under different culture conditions shows that Treg cells induced in the presence of RAPA show the most potent suppression of effector T cells.

## Materials and methods

### Blood donors and samples

The study was approved by the Institutional Ethics Committee from Clinica Las Condes (July 27^th^. 2011) and by the Ethics Committee of the funding agency FONDECYT (N°018/FONDECYT/Medicina G1/0381, May 24^th^.2012). Written informed consent was obtained from all donors. Peripheral blood was collected from healthy volunteers aged 18 to 40 years. Donors with any known current disease or receiving pharmacological therapy were excluded from the study.

### Naïve T cell isolation

Peripheral blood mononuclear cells (PBMCs) were isolated from 300 mL of whole blood from healthy donors after centrifugation on a Ficoll-Hypaque density gradient (GE Healthcare, Uppsala, SE). CD4+CD45RA+ naïve T cells (Tn) were isolated by negative selection, utilizing a naïve CD4+ T Cell Isolation Kit II with a QuadroMACS separator unit (MiltenyiBiotec, Bergisch Gladbach, DE) according to the manufacturer’s instructions. The purity of the isolated CD4+CD45RA+ cells was evaluated with a BD FACSCanto^TM^ II flow cytometer and FACSDiva software (BD Biosciences, San Jose, CA, USA). Purity was above 97%, and CD4+CD25+FOXP3+ cells were below 2% for all donors.

### Generation and phenotype analysis of polyclonal CD4+CD25+FOXP3+ T Cells

Freshly isolated CD4+CD45RA+ naïve T cells were stimulated with beads conjugated with anti-human anti-CD3/CD28 antibodies at a ratio of one bead to 10 cells (Invitrogen, Carlsbad, CA, USA) in AIM-V serum-free medium containing HEPES buffer (10 mM), sodium pyruvate (1 mM), glutamax (2 mM), non-essential amino acids (1 mM), penicillin and streptomycin (complete medium) (Invitrogen, Carlsbad, CA, USA). This medium was supplemented with different combinations of IL-2 (100 U/mL), TGF-β1 (5 ng/mL) (carrier-free; R&D Systems, Minneapolis, MN, USA), and a range of atRA concentrations (1nM—10 μM) (Sigma-Aldrich, St. Louis, MO, USA) and/or RAPA (100 nM) (EMD Millipore, MA, USA), as indicated. Cells were stimulated under these conditions for 5 days in 96 round-bottom well culture plates, incubated at 37°C in a humidified 5% CO2, and 95% atmospheric air.

After that, cells were washed with Stain Buffer (FBS, Becton Dickinson, New Jersey, NY, USA) and labelled with PerCP-labelled mouse anti-human CD4 (Clone SK3) and APC-labelled mouse anti-human CD25 (Clone 2A3) in addition to PE-labelled mouse anti-human CD45RA (Clone HI100), CXCR4 (Clone 12G5), CD62L (Clone DREG-56), or CCR9 (Clone 112509) (BD Biosciences, San Jose, California USA), or PE-labelled rat anti-human β-7 (Clone FIB504) (Biolegend, San Diego, California USA). The samples were incubated in the dark for 30 minutes on ice and washed twice. Thereafter, the cells were recovered, permeabilized with human FOXP3 Buffer Set (BD Biosciences, San Jose, California USA), washed and stained with an Alexa Fluor 488-labeled mouse anti-human FOXP3 antibody (Clone 259D/C7) according to the manufacturer’s instructions (BD Biosciences, San Jose, California USA). In parallel, staining with isotype-matched nonspecific antibodies recommended by the manufacturer was included. The percentage of Treg cells generated and the expression of surface molecules were determined by flow cytometry, using a FACSCanto II and the FACSDiva software (BD Biosciences, San Jose, CA, USA). Dead cells were excluded from the analysis utilizing LIVE/DEAD Fixable Dead Cell Violet Stain Kit, according to the manufacturer’s instructions (Life Technologies GIBCO®, Carlsbad, CA, USA).

Absolute counts were indirectly obtained from the percentage of life CD4+CD25+FOXP3+ cells and total counts of viable cells obtained from the trypan blue exclusion assay (Live CD4+CD25+FOXP3+ cells x 100/Trypan blue total viable cells).

MFI normalization was performed individually for each donor. Calculations were performed utilizing following formula: normalized MFI (nMFI) of the test sample = MFI of the test sample x100/highest MFI value for the experiment.

### Methylation analysis of CNS2

Methylation analyses were performed on sorted peripheral natural Treg cells (nTreg) as well as on Treg induced from naïve T (Tn) cells obtained from male donors and cultured under different conditions (see Results).

Peripheral nTreg cells utilized as controls were isolated following a two-step procedure. First, using a CD4+CD25+CD127dim/- regulatory T cell isolation kit II from Miltenyi Biotec (Miltenyi Biotec, Bergisch Gladbach, DE) according to the manufacturer’s instructions. Next, intracellular FOXP3 staining was performed, as previously described and the CD4+CD25+FOXP3+ T cells were sorted using a BD FACSCanto^TM^ cell sorter. These cells were utilized for comparison of the methylation status of the induced Treg cells.

Methylation analyses of iTreg were also performed on FACS-sorted Treg cells. Induced Treg generated under the different conditions as indicated above were permeabilized with human FOXP3 Buffer Set (BD Biosciences, San Jose, California USA), gated on the CD4+ marker and the CD25high / FOXP3+ cells were identified and sorted.

Unless otherwise specified, the sorted subsets reached >97% purity based on CD4+CD25highFOXP3+ staining. DNA extraction, bisulphite conversion, and pyrosequencing analysis were conducted by EpigenDx (Worcester, MA, USA). Eleven CpGs in the first intron of the *FOXP3* gene were analyzed (-2376 to -2263 from ATG, ENST00000376207). Assay ID: ADS783FS1 and ADS783FS2 [44,51].

### In vitro suppression assay

Responder T cells (Tresp) (CD4+CD25- cells) were obtained by immunomagnetic negative selection of CD4+ lymphocytes from PBMCs obtained from 50 ml of peripheral blood of healthy donors, utilizing a CD4 T cell isolation kit II (MiltenyiBiotec, Bergisch Gladbach, DE). After that, the CD4+ lymphocytes were depleted of CD25+ cells, utilizing the CD25 MicroBeads II separation kit (MiltenyiBiotec, Bergisch Gladbach, DE). The isolated CD4+CD25- cells were characterized by flow cytometry and stained with CellTrace Violet (Life Technologies GIBCO®, Carlsbad, CA, USA) according to the manufacturer’s instructions. Also, autologous Treg cells, generated under different conditions, as described, were harvested after 5 days, counted and re-suspended at 1x10^6^ cells/mL in sterile PBS (Life Technologies GIBCO®, Carlsbad, CA, USA). After that, the CD4+CD25HighCD127- Treg cells (anti-CD127-PE: clone HIL-7R-M21, BD Biosciences) were FACS sorted (reaching over 98% of purity). T cells were identified by their forward scatter (FSC) vs. side scatter (SSC) profile. Doublets were excluded and the CD25highCD127- cells identified within the CD4 gate were sorted. After sorting, the obtained cells were rested for 12 hours, and then washed with fresh medium. Afterwards, the cells were co-cultured with autologous responder T cells (CellTrace Violet-labelled cells) at different Treg:Tresp cell ratios, ranging from 1:4 to 1:64 in 96-well round-bottom plates previously coated with 10 μg/mL of anti-CD3 antibody (clone OKT3, LEAF grade; Biolegend®, San Diego, CA, USA). The assay was performed in complete RPMI 1640 medium (Life Technologies GIBCO®, Carlsbad, CA, USA containing 10% pooled AB serum, 25 mM HEPES, 1 mM sodium pyruvate, glutamax (2mM), non-essential amino acids (1mM) and penicillin/streptomycin) plus 10 μg/mL of soluble anti-CD28 (clone CD28.2, LEAF grade; Biolegend®). Each well contained a constant number of Tresp cells (1.0x10^5^ cells/well) in a final volume of 200 μl. To exclude non-specific inhibition of proliferation due to differences in cell density, we performed co-cultures including cell trace violet labeled and non-labeled Tresp cells, in the same ratios as the tested induced Treg cells (density control) [52].

The plates were incubated at 37°C in a humidified 5% CO2 for 4 days. Thereafter, the cells were collected and the violet stain dilution was analyzed by flow cytometry gating on the CellTrace Violet+ lymphocytes. Results were reported as percentage of suppression = 100 –(100 x % of proliferating Tresp cells with generated Treg cells/% of proliferating Tresp cells in the cell density control)) [52–54].

### Statistical analysis

Statistical analysis was performed using GraphPad Prism 5.0 software (La Jolla, CA, USA). Data were calculated as the median with 25th and 75th percentiles and maximal/minimal values. Statistical significance was determined by two-tailed paired t tests, two-way repeated measures ANOVA followed by Tukey’s or by Sidak’s Multiple-Comparison post-hoc test as indicated in the legends.

## Results

### Effects of atRA on the induction of Treg cells

**Fig 1:**
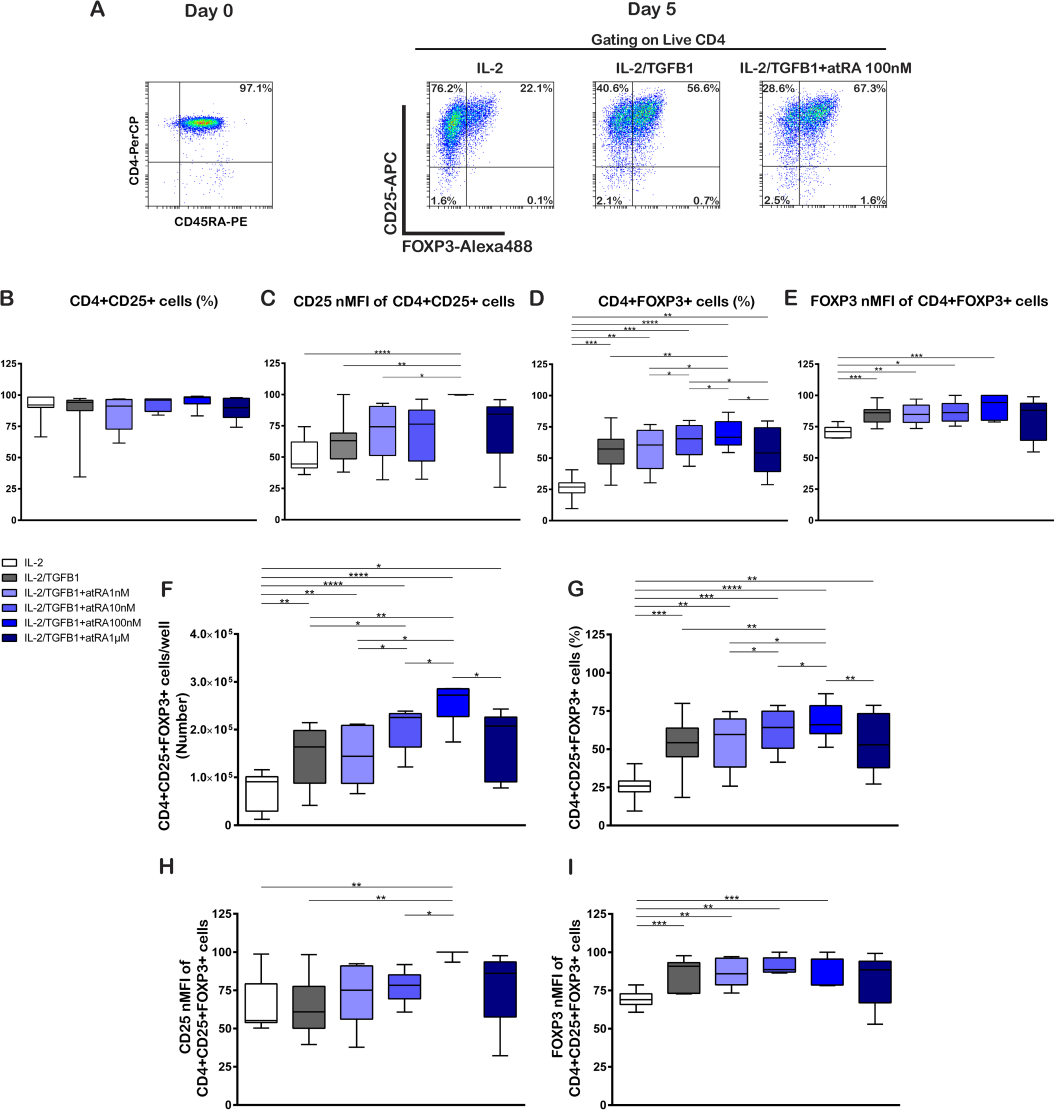
atRA enhances the generation of TGF-β1/IL2-induced Treg cells from naive T cells. Purified naive T cells (CD4^+^CD45RA^+^) were stimulated on day 0 with anti-CD3/CD28 coated beads in the presence of IL-2 (100U/mL), TGF-β1 (5ng/mL), and different concentrations of atRA. Cell phenotype was analyzed by flow cytometry on day 5. (A) Representative dot plots of one donor show the expression of CD45RA+ on the isolated naive CD4 T cells on day 0, and the expression of CD25 and FOXP3 by the generated cells under three different culture conditions on day 5. (B) Percentage of CD25+ cells. (C) CD25 nMFI of CD25+ cells. (D) Percentage of FOXP3+ cells. (E) FOXP3 nMFI of CD25+ cells. (F) Number of CD4+CD25+FOXP3+ Treg cells. (G) Percentage of CD4+CD25+FOXP3+ Treg cells. (H) CD25 nMFI expression of CD4+CD25+FOXP3+ Treg cells. (I) FOXP3 nMFI of CD4+CD25+FOXP3+ Treg cells. Cells were cultured for 5 days in presence of IL-2 (100U/mL), TGF-β1 (5ng/mL) and different concentrations of atRA as shown. Statistical significance was determined by two-tailed paired t tests. *p < 0.05; **p < 0.01; ***p < 0.001; ****p < 0.0001 for n = 7–8 donors in independent experiments. Gating strategy and representative dot plots are shown in S1 Fig.

Due to the high number of Treg cells needed to obtain clinical effects, our first aim was to determine the optimal atRA concentration required to induce the uppermost number of Treg cells in short time cultures. Tn cells were selected based on the simultaneous expression of CD4+ and CD45RA+. Treg cells were defined based on the concomitant expression of the canonical markers CD4+, CD25+, and FOXP3+. After isolation, the starting cell population contained over 97% Tn cells, and Treg cells were below 2%. Fig 1A and S1 Fig show representative plots of the analysis performed. Polyclonal activation and culture of Tn cells in presence of IL-2 (100 U/mL) and TGF-β1 (5 ng/mL), without atRA or with atRA at varying concentrations (1nM, 10 nM, 100 nM and 1 μM) resulted in a very similar percentage of total CD4+ cells expressing CD25 (CD4+CD25+) in all conditions (Fig 1B, S1 Table). Higher atRA concentrations (10 μM) correlated with a cell death of nearly 100%. Moreover, the expression of CD25 per cell (measured as normalized mean fluorescence intensity, nMFI) in CD4+CD25+ cells treated with IL-2 had a median of 44.5%. The addition of TGF-β1 showed 63.1% which increased significantly in cells cultured with TGF-β1 plus atRA 100 nM (100.0%, p = 0.0026). There were no significant differences when atRA in other concentrations was added to IL-2 + TGF-β1 (Fig 1C, S1 Table).

The proportion of CD4 cells expressing FOXP3 (CD4+FOXP3+) was significantly higher in both IL-2/TGF-β1 and IL-2/TGF-β1 + atRA treatments in comparison to IL-2 alone (Fig 1D, S1 Table). As the atRA concentration increased to 100 nM, we observed a higher percentage of CD4+FOXP3+ cells, whereas at higher concentration (1 μM) we found a lower percentage of CD4+FOXP3+ cells in comparison with atRA 100 nM (p = 0.0134) (Fig 1D).

The FOXP3 nMFI showed small differences between the different culture conditions with the highest values in presence of IL-2/TGF-β1+atRA 100 nM (Fig 1E, S1 Table).

CD4+FOXP3+ cells showed a tendency towards a higher percentage and CD25 nMFI compared to CD4+FOXP3- cells. This difference was enhanced in cells cultured with IL-2, TGF-β1, and atRA 100 nM (S1C1 and S1C2 Fig). Comparing FOXP3 expression in CD4+CD25+ and CD4+CD25- cells, it was observed that in all culture conditions, CD25 positive cells expressed FOXP3 at a higher percentage and nMFI than CD25-negative cells (S1 C3 and C4).

When we analyzed the Treg (CD4+CD25+FOXP3+) cell population, we observed that the cultures in the presence of IL-2 attained a yield of 9.1x10^4^ Treg cells per well representing 25.9% of all CD4+ cells after 5 days (Fig 1F and 1G). The addition of TGF-β1 and TGF-β1 plus atRA to IL-2 treatment increased the yield of Treg cells (S1 Table) significantly. Similarly as shown previously in the percentage of CD4+FOXP3+ cells we observed that the amount of CD4+CD25+FOXP3+ Treg cells increased up to an atRA concentration of 100 nM, whereas a higher concentration (1 μM) was related to a lower yield (Fig 1F). A similar increase was observed in the Treg cells considered as a percentage of the total recovered cells (Fig 1G, S1 Table).

MFI analysis of CD25 showed that atRA 100 nM induced a significantly higher expression of CD25 in the Treg cell population when added to IL-2/TGF-β1 treatment or when compared to IL-2/TGF-β1 with lower atRA concentrations (Fig 1H, S1 Table). Further, we observed a lower nMFI of FOXP3 in the presence of IL-2 alone in comparison with IL-2/TGF-β1 and IL-2/TGF-β1 with atRA 1–100 nM, showing the same trend as the CD4+FOXP3+ cell percentage, nMFI and Treg cells yield analysis. (Fig 1I).

Taken together, the results shown are consistent with a higher yield of Treg cells in cultures supplemented with atRA 100nM, which is related to a higher percentage of FOXP3 expressing cells. Further, the FOXP3 positive cells express higher levels of CD25 than FOXP3 negative cells, a fact that may have functional consequences, as discussed below.

### Effects of RAPA alone or with atRA on the induction of Treg cells

It has been reported that RAPA works in synergy with TFG-β1 up-regulating FOXP3+ Treg cells [55]. Accordingly, we evaluated the effects of RAPA in our experimental setting.

**Fig 2:**
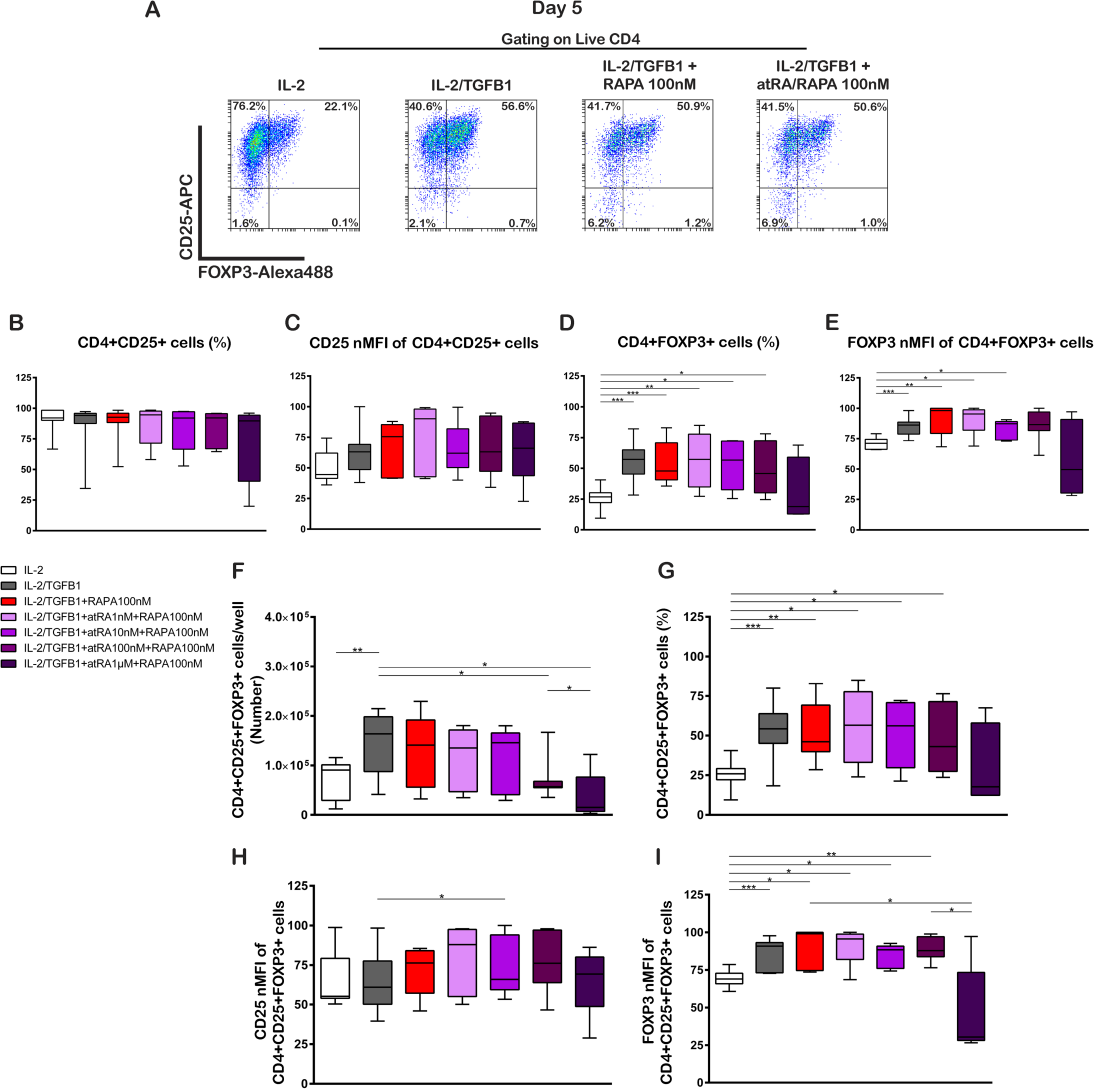
RAPA alone or in combination with atRA does not enhance the generation of Treg cells from naive T cells in short-time cultures. Naïve CD4^+^ T cells were obtained and stimulated as in Fig 1, in the presence of IL-2 (100U/mL), TGF-β1 (5ng/mL), RAPA (100ng/mL) and different concentrations of atRA as shown. The phenotype of the cultured cells was analyzed by flow cytometry on day 5. (A) Representative dot plots of one donor show the expression of CD25 and FOXP3 by the generated cells under four different culture conditions on day 5. (B) Percentage of CD25+ cells. (C) CD25 nMFI of CD25+ cells. (D) Percentage of FOXP3+cells. (E) FOXP3 nMFI of CD25+ cells. (F) Number of CD4+CD25+FOXP3+ Treg cells. (G) Percentage of CD4+CD25+FOXP3+ Treg cells. (H) CD25 nMFI of CD4+CD25+FOXP3+ Treg cells. (I) FOXP3 nMFI of CD4+CD25+FOXP3+ Treg cells. Statistical significance was determined by two-tailed paired t tests. *p < 0.05; **p < 0.01; ***p < 0.001; ****p < 0.0001 for n = 7–8 donors in independent experiments. Gating strategy is shown in S1 Fig and representative dot plots are shown in S2 Fig.

Fig 2A and S2A Fig show representative plots of the analyses performed. Polyclonal activation and culture of Tn cells in the presence of IL-2 (100 U/mL), IL-2 with TGF-β1 (5 ng/mL) and IL-2 with TGF-β1 plus RAPA (100 nM) as well as RAPA combined with atRA at varying concentrations (1nM, 10 nM, 100 nM and 1 μM) resulted in a very similar percentage of CD4+CD25+ cells in all conditions (Fig 2B, S1 Table). The CD25 nMFI on CD4+CD25+ cells also showed no significant changes under the different culture conditions (Fig 2C, S1 Table). Cultures of Tn with IL-2/TGF-β1 in the presence of RAPA alone or RAPA with atRA in varying concentrations showed no increase in the proportion of FOXP3 expressing cells in comparison to IL-2/TGF-β1 (Fig 2D, S1 Table). The FOXP3 nMFI showed an increase in the presence of IL-2/TGF-β1 and IL-2/TGF-β1 with RAPA in comparison with IL-2 alone. The addition of atRA 1–10 nM to IL-2/TGF-β1 with RAPA did not increase further the nMFI of FOXP3. Higher atRA concentrations, 100nM and 1uM, did not show differences with IL-2 alone (Fig 2E, S1 Table). A comparison of CD4+FOXP3+ and CD4+FOXP3- cells showed no differences in the percentage of CD25+ cells (S2B1 Fig), while FOXP3 positive cells showed significantly higher CD25 nMFI than FOXP3-negative cells (S2B2 Fig), independent of the culture conditions. Furthermore, the expression of FOXP3 was higher regarding the percentage and nMFI in CD4+CD25+ cells that in CD4+CD25- cells (S2B3 and S2B4 Fig, S1 Table).

When we analyzed the number of CD4+CD25+FOXP3+ cells, we observed a higher yield on IL-2/TGF-β1 cultured cells in comparison with IL-2 alone. The addition of RAPA did not increase further the number of Treg cells. On the contrary, the addition of RAPA with atRA in high concentrations (atRA100 nM and 1μM) lowered the number of Treg cells obtained in contrast with IL-2/TGF-β1 treatment (Fig 2F, S1 Table).

In a similar fashion than CD4+FOXP3+ cells, the percentage of CD4+CD25+FOXP3+ cells showed an increase when TGF-β1, RAPA or RAPA plus atRA 1–100 nM were added to IL-2 alone (Fig 2G, S1 Table). The CD25 nMFI showed only minimal differences in the different conditions (Fig 2H, S1 Table). The FOXP3 nMFI observed for IL-2 alone was lower than with IL-2/TGF-β1 and in comparison to IL-2/TGF-β1 with RAPA. The combinations of IL-2/TGF-β1 plus RAPA with atRA 1–100 nM also showed a significant increase in comparison to IL-2 alone (Fig 2I, S1 Table). The addition of atRA 1 μM decreased the FOXP3 nMFI of CD4+CD25+FOXP3+ cells compared to IL-2/TGF-β1 with RAPA and compared to the atRA 100 nM treatment (Fig 2I, S1 Table).

Collectively, these results showed that under these experimental conditions, RAPA alone and RAPA with atRA had no additional effect on the number and percentage of Treg cells in comparison with IL-2/TGF-β1.

### Activation and homing receptors: The effects of atRA and RAPA

**Fig 3:**
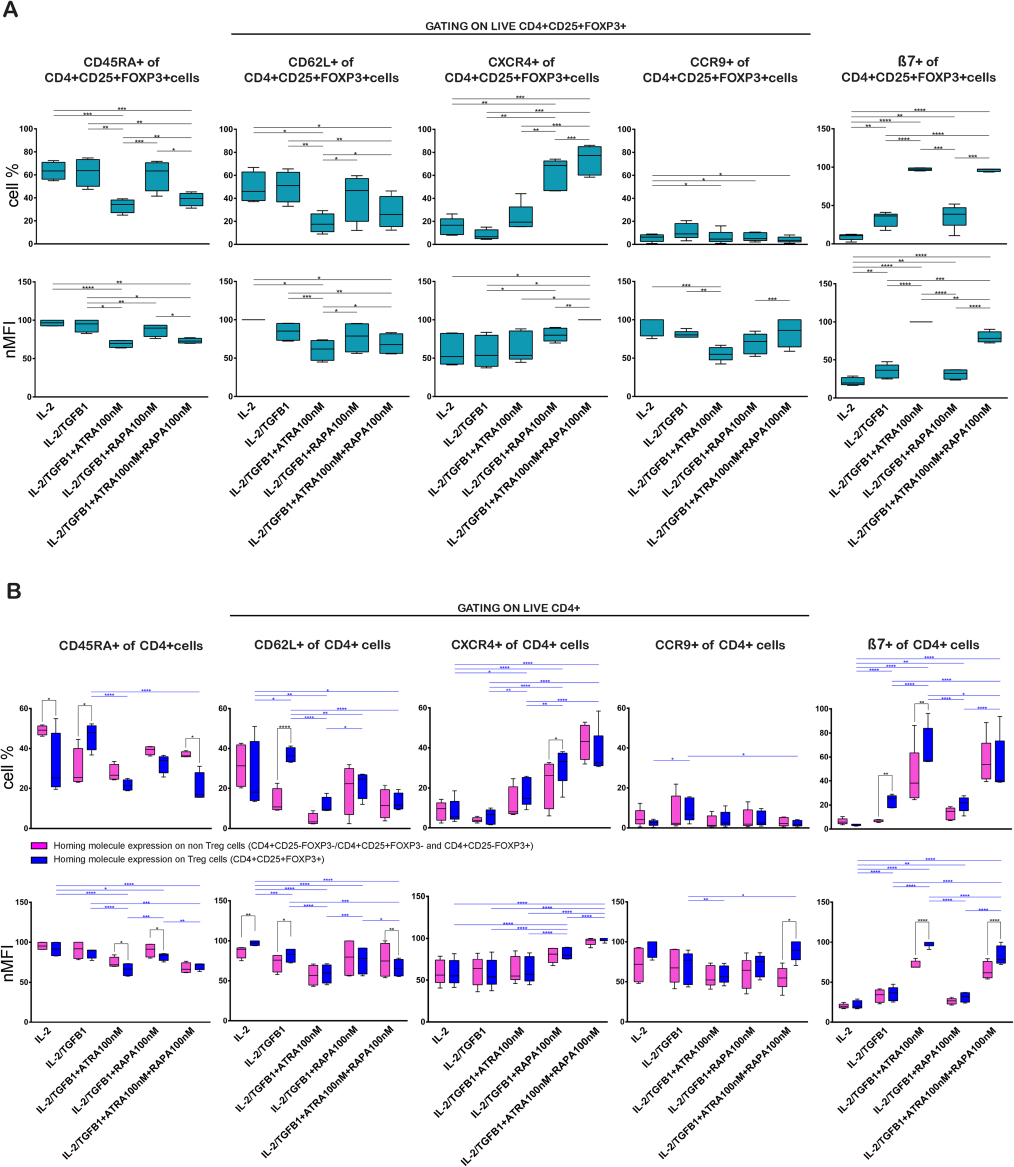
TGFβ1, atRA and RAPA have diverse effects on the induction of CD45RA, CD62L, CXCR4, CCR9 and β7 expression on Treg cells. Expression of CD45RA, CD62L, CXCR4, CCR9 and β7 on the CD4+CD25+FOXP3+ Treg cells induced with IL-2 (100U/mL), TGF-β1 (5ng/mL), RAPA (100ng/mL) and atRA (100nM). (A) Percentage and nMFI of homing molecules expressed by CD4+CD25+FOXP3+ Treg cells. Statistical significance was determined by two-tailed paired t tests. (B) Percentage and nMFI comparison of the homing molecules expression on Treg and non-Treg cells. The CD4+CD25+FOXP3+ Treg cells are shown in blue bars, cells either CD25- or FOXP3- or both are labeled as non-Treg cells and are shown in magenta bars. Gating strategy as shown in S3 Fig. Significance was calculated by two-way repeated-measures ANOVA and Sidak's post-test. *p < 0.05; **p < 0.01; ***p < 0.001; ****p < 0.0001. n = 4–5 donors in independent experiments. Gating strategy and representative dot plots of all conditions are shown in S3 Fig.

Representative plots of the analyses performed are shown in S3 Fig. To distinguish naïve from activated T cells, the expression of CD45RA and CD62L was analyzed on induced Treg cells (CD4+CD25+FOXP3+) as well as in total CD4+ T cells (Fig 3, S3 Fig and S1 Table). Treg cells induced in the presence of IL-2 alone and IL-2/TGF-β1 showed the highest CD45RA percentage and level expression on CD4+CD25+FOXP3+ Treg cells in comparison to Treg cells induced by the other treatments (Fig 3A, S1 Table). The addition of RAPA to the cultures showed no important changes of CD45RA percentage and level expression. Induced Treg cells showed the lowest values of CD45RA in terms of cell percentage and expression level when atRA was added to the cultures.

The expression of CD62L is characteristic of naïve T cells and central memory T cells, representing a homing molecule to secondary lymphoid tissues. In general, the expression of CD62L measured in terms of percentage and MFI followed a pattern similar to CD45RA on CD4+CD25+FOXP3+ Treg cells. The cultures in the presence of IL-2/TGF-β1 correlated with the highest percentage of CD62L, reaching 51.1% of the Treg cells. Moreover, the expression level of CD62L on Treg cells showed a similar pattern as CD45RA with the highest CD62L expression on Treg cells treated with IL-2 alone, reaching a nMFI of 100.0%. The addition of RAPA showed no changes in the CD62L in terms of percentage and MFI in comparison to IL-2 alone and IL-2/TGF-β1. Again, similar to the results of CD45RA expression, the presence of atRA was related to the lowest proportion and expression level of CD62L (Fig 3A, S1 Table).

We observed a trend towards a down-regulatory effect of atRA on the expression of CD45RA and CD62L even in the presence of RAPA.

The homing potential of the generated Treg cells was also investigated by the expression of molecules that modulate homing to the gut (β7, CCR9), and bone marrow (CXCR4), which are crucial in the context of developing a strategy to facilitate bone marrow and organ transplantation.

The presence of RAPA induced an increase in the percentage and expression level of CXCR4 in comparison with IL-2 TGF-β1, which increased further in the presence of RAPA with atRA (Fig 3A, S1 Table). The nMFI of CXCR4 showed no significant rise in the presence of RAPA in comparison with IL-2, but increased in the presence of RAPA with atRA (Fig 3A, S1 Table). The atRA addition to IL-2/TGF-β1 showed no significant increase in percentage and expression of CXCR4 on the Treg cells (Fig 3A S1 Table).

The expression of CCR9 was low in all studied conditions. The addition of atRA, RAPA or their combination to the basic culture conditions decreased further the percentage of CCR9 expression on Treg, in comparison with IL-2/TGF-β1, particularly in the presence of the combined treatment with both molecules. Moreover, the CCR9 nMFI showed the lowest value for IL-2/TGF-β1 plus atRA 100 nM in comparison to IL-2 and IL-2/TGF-β1 (Fig 3A, S1 Table).

Remarkably, the presence of atRA, alone or in combination with RAPA, increased the percentage of β7 obtained with IL-2/TGF-β1 from 36.4% to 97.3% and 97.1% of the Treg, and the nMFI increased from 36.4% to 100.0% and 78.2%. RAPA without atRA showed a similar β7 percentage and nMFI in comparison to IL-2/TGF-β1. The addition of RAPA to IL-2/TGF-β1 did not produce important disparities. (Fig 3A, S1 Table).

The comparison between the non-Treg and Treg cells for each treatment showed that non-Treg cells tend to have a higher percentage and expression of CD45RA in all treatments with the exemption of IL-2/TGF-β1 treatment (Fig 3B, S1 Table). Analogous to CD45RA, the expression of CD62L increased on Treg cells in the presence of IL-2/TGF-β1, a difference which reversed in the presence of AtRA and RAPA (Fig 3B, S1 Table). Analysis of the differential expression of these activation and homing molecules at the end of the culture period showed that CXCR4 and CCR9 were similarly expressed in Treg cells as well as non-Treg cells (Fig 3B, S1 Table).

atRA was related to an increase of β7 on Treg as well as non-Treg cells. Remarkably, this effect was more pronounced on Treg cells than non-Treg cells (Fig 3B, S1 Table).

### Effects of atRA and RAPA on the methylation of CNS2

**Fig 4:**
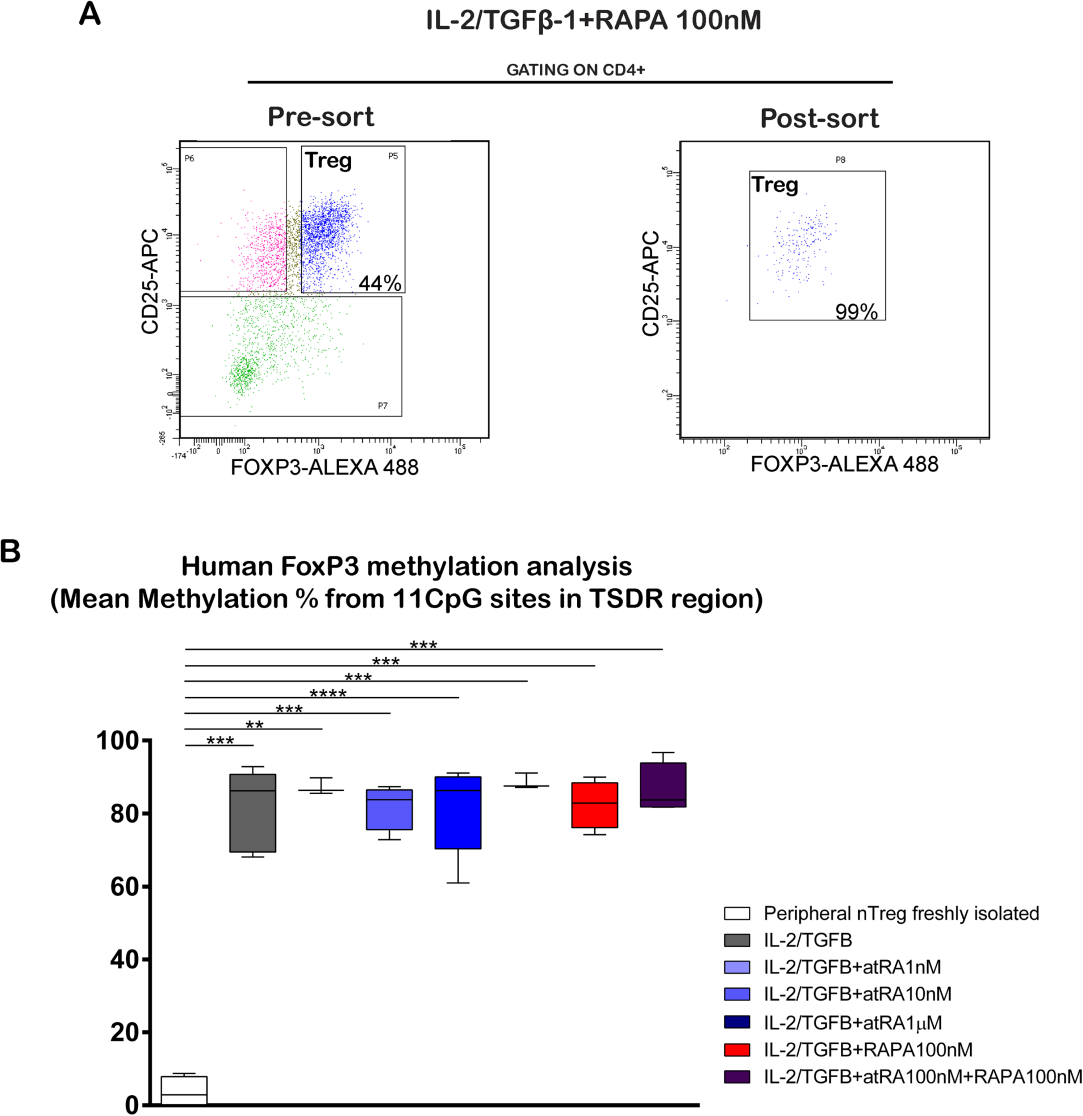
Treg cells induced in the presence of atRA and/or RAPA are highly methylated in the CNS2 region. Treg were generated as shown in Fig 1. After 5 days, CD4+CD25+FOXP3+ Treg cells obtained under the different treatments as well as fresh, non cultured Treg cells were sorted. The methylation of the CNS2 region was analyzed by bisulphite conversion and pyrosequencing (EpigenDx). (A) Representative dot blots of the cells obtained before and after sorting. (B) Methylation percentage of the CD4+CD25+FOXP3+ Treg cells. The methylation of each sample represents the mean value of the 11 CpG sites analyzed. Methylation of the specific CpG sites is shown in S4 Fig. Statistical significance was determined by two-tailed paired t tests. *p < 0.05; **p < 0.01; ***p < 0.001; ****p < 0.0001. n = 3–6 donors in independent experiments.

The methylation of the CNS2 region of the *FOXP3* gene in induced Treg cells was assessed. Methylation on Treg cells isolated from peripheral blood was also evaluated and utilized for comparison. Induced Treg cells and Treg cells isolated from peripheral blood were sorted as described, showing an enrichment of CD4+CD25+FOXP3+ Treg cells above 98% (Fig 4A). A scheme of the *FOXP3* gene with the studied sequences is shown in S 4A.

In Treg cells isolated from peripheral blood, we observed a demethylated pattern with a methylation range of 0.5 to 8.8% in different donors. In contrast, induced Treg cells cultured in the presence of IL-2/TGF-β1, RAPA or atRA showed a highly-methylated pattern, which varied between 85.9 and 88.6%. The mean value of the 11 CpG sites analyzed showed no differences between the diverse culture conditions (Fig 4B). Furthermore, the methylation of each CpG showed a similar pattern between each single CpG’s, and no differences between the different culture conditions (S4B Fig and S1 Table). In conclusion, Treg cells induced in the presence of atRA, RAPA or both show a highly-methylated pattern of the CNS2, with a similar methylation of the single CpG’s analyzed.

### Impact of atRA and RAPA on the functionality of the generated Treg cells

**Fig 5:**
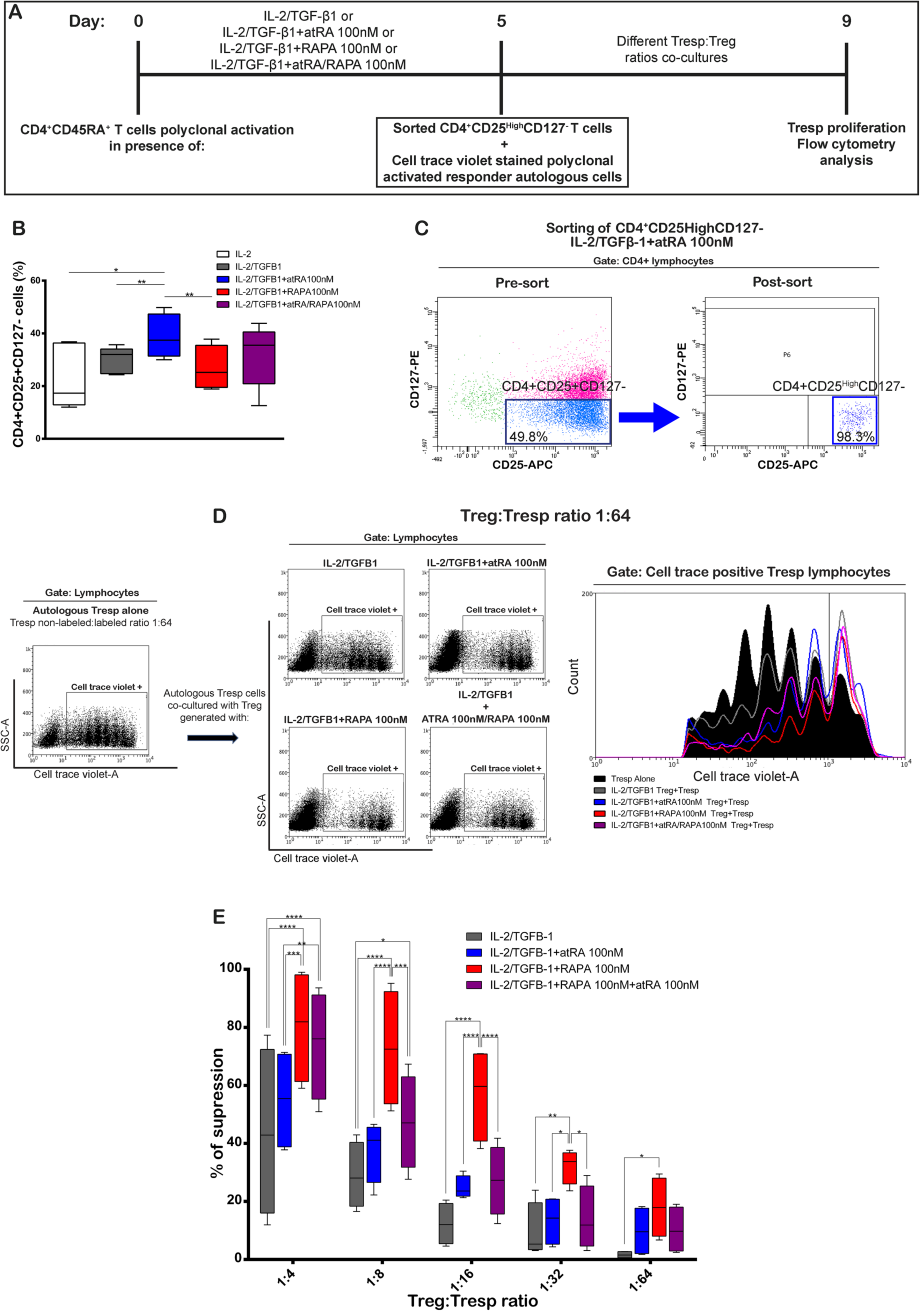
Treg cells generated with IL-2-TGFβ-1 and RAPA have higher suppressive capacity than cells generated with IL-2/TGFβ-1 or with IL-2/TGFβ-1 plus atRA. CD4+CD25- responder T cells were isolated from PBMC from healthy donors, stained with CellTrace Violet, activated with plate bound anti-CD3 and soluble anti-CD28. (A) Cells were co-cultured with different ratios of FACS sorted CD4+CD25highCD127- Treg cells generated under the indicated conditions. Responder T cells and Treg cells were obtained from the same donors. Co-cultures were maintained for 4 days, harvested and FACS analyzed for CellTrace Violet stain. (B) Percentage of CD4+CD25+CD127- cells induced by the different treatments. Statistical significance was determined by two-tailed paired t tests for n = 5 donors in independent experiments. (C) Representative dot plots of the Treg cells induced with IL-2/TGFβ-1 + atRA 100 nM before and after sorting. Additional information on the expression of CD25 and FOXP3 on the sorted cells is shown in S5B Fig (D) Representative dot plots and histogram of the CellTrace Violet-based proliferation assay after 4 days of culture. Data obtained from responder cells alone and responder cells with Treg cells induced under different listed treatments at ratio 1/64 are shown. (E) Suppression activity calculated as percentage as described in “Materials and Methods” for Treg cultured under the shown conditions. Significance was calculated by two-way repeated-measures ANOVA and Tukey's post-test. *p < 0.05; **p < 0.01; ***p < 0.001; ****p < 0.0001 for n = 4 donors in independent experiments.

To compare the suppressive activity of the CD4+CD25+CD127^-^ Treg cells generated in the presence of IL-2/TGF-β1, atRA, RAPA, or their combination, Treg cells were induced, isolated by flow sorting, co-cultured at different ratios for 4 days with Cell Trace Violet stained autologous responder T cells, and evaluated by the proliferation of the Tresp cells (Fig 5A).

Treg cells were induced in the presence of IL-2/TGF-β1, IL-2/TGF-β1 with atRA 100 nM, IL-2/TGF-β1 with RAPA 100 nM and IL-2/TGF-β1 with atRA 100 nM and RAPA 100 nM. In accordance with the previous results, cultures in the presence of IL-2/TGF-β1 with atRA 100 nM showed the highest percentage of Treg cells (Fig 5B). Representative plots of the cells obtained before and after sorting are shown in Fig 5C and S5A and S5B Fig).

The Treg cells obtained after FACS sorting showed enrichment for CD4+CD25+CD127- above 98% (S5 A), and comprised 57–88% FOXP3+ cells (S5 B).

Representative plots of the proliferation of Tresp cells alone and in co-culture with Treg cells at a ratio Treg:Tresp 1:64 is shown in Fig 5D. We observed a robust proliferation of the Tresp cells when cultured in the absence of Treg cells (ranges between 72 and 93.9% of proliferating cells, based on cell trace dye). On the other hand, depending on the Treg cells:Tresp ratio and the culture conditions of the induced Treg cells, the proliferative capacity of the Tresp cells decreased substantially.

Functional comparison of Treg cells induced under different culture conditions showed that the highest suppressive activity was achieved by Treg cells obtained from cultures with IL-2/TGFβ-1 plus RAPA. The percentage of suppression by Treg cells cultured in the presence of IL-2/TGFβ-1 plus RAPA was higher than cells cultured with IL-2/TGFβ-1 alone or with atRA, which was more pronounced at Treg cells:Tresp ratio of 1:16 (Fig 5E, S1 Table). Treg cells generated in the presence of IL-2/TGFβ-1+atRA showed higher suppression than IL-2/TGFβ-1 alone, but lower suppression than IL-2/TGFβ-1+RAPA. Treg cells induced in the presence of IL-2/TGFβ-1+RAPA+atRA showed an intermediate suppression result. Taken together, in our experimental conditions, Treg cells induced in the presence of RAPA did not increase the generation of Treg cells but increased the suppressive function of the induced Treg cells.

## Discussion

One of the main limitations to an effective adoptive therapy with Treg cells is the high number of cells needed and the difficulties related to achieving this goal. The most remarkable clinical report on the infusion of expanded Treg cells showed benefits in treating graft versus host disease with 3–100*10^6^ cells/Kg [56]. Therefore, obtaining a high number of Treg cells is of the utmost importance.

IL-2 and TGF-β1 are crucial to allow for the differentiation of naïve T cells into regulatory T cells. Of note, IL-2 alone was able to induce Treg cells to 25.9% in our setting, while TGF-β1 increased Treg cells to 54.2%. Both cytokines activate the crucial transcription factors, STAT5 and Smad2/3, which are essential elements for expression of the gene *FOXP3*. These results are in accordance with Zheng *et al*.[57], who showed a mutual dependence of both cytokines in the induction of Treg cells. The importance of IL-2 is further highlighted by the crucial role of its receptor in the induction and functional activity of Treg *in vivo* [58]. It may be surprising that IL-2 alone can enhance the expression of FOXP3 up to 25%. However other authors utilizing a similar [59] or a different methodology [60] have found similar results. It should also be considered that activated human T cells, differently from mice, may transiently express FOXP3 without acquiring regulatory function [61,62].

atRA has also been added to enhance the generation of human Treg cells; however, no systematic dose response assays have been reported. For example, Wang *et al*. [63], Dons *et al*. [64], and Schmidt *et al*. [39] reported the induction of human Treg cells with atRA 10 nM, while Lu *et al*., [59], and Ma *et al*., [65] utilized 100 nM with the same aim. Further, Golovina *et al*. [66], reported on the expansion of human natural Treg cells with 10 nM atRA under serum-free conditions and 10 μM for serum-containing conditions.

Here, we report on the generation of Treg under conditions of polyclonal activation with anti-CD3 and anti-CD28 loaded beads, serum free media conditioned with IL-2 and TGF-β1 and a broad range of concentrations of atRA. Our results showed that the best results regarding number and percentage of CD4+CD25+FOXP3+ cells were obtained with 100 nM of atRA; whereas lower levels showed no difference with IL-2 and TGF-β1 alone, 10 nM enhanced the number of Treg cells, but it did not reach statistical significance, possibly due to the limited sample size. Higher concentrations (1.0 μM), showed a decrease in both the number and proportion of Treg cells, which was related to mortality above 80%. The results shown suggest that atRA has Treg cells inducing properties in a wide range of concentrations, whereas the highest effect was obtained with 100 nM. It must be considered that the effects with the concentrations reported here may also depend on the kind of activation. For comparison, the type of activation in our study was similar to that employed by Ma *et al*., [65] utilizing beads anti-CD3/CD28 at a ratio 1 bead to 10 cells and Lu *et al*. [50] also using anti-CD3/CD28 beads at a ratio 1 bead to 5 cells, though with longer culture times. In contrast, Wang *et al*. [63], and Schmidt *et al*. [39] utilized plate-bound anti-CD3 antibodies and soluble anti-CD28 antibodies. A direct comparison of beads with plate-bound antibodies would be valuable to compare the efficiency of the different activation methods. A comparison of the FOXP3 expression of induced Treg cells versus natural Treg cells has been performed by *Schmidt et al* [39], showing that a higher number of unstimulated natural Treg cells express FOXP3 in comparison to induced Treg cells. However, the difference between unstimulated natural Treg cells and induced Treg cells cultured with IL-2/TGF-β1 and atRA was only 16%.

The increased number of Treg cells observed with IL-2, TGF-β1, and atRA 100 nM was mainly related to an increased number and percentage of FOXP3+ cells. Suitable mechanisms for the enhanced FOXP3 expression involve up-regulation of SMAD3 [67], up-regulation of ERK1/2 [50], and RAR alpha 2 and epigenetic modifications [26]. For a detailed discussion on the mechanisms, we refer to a recent review from the group led by Noelle [68].

It was observed that atRA did not increase the total number and proportion of CD25+, T cells, although increasing the MFI of CD25 particularly in the FOXP3-positive cells. It has been shown that the expression level of CD25 on Treg cells, measured as the fluorescence intensity, affects the ability to bind IL-2 and transduce IL-2 signaling [69]. Moreover, it has also been shown that low CD25 expression and lower IL-2 signaling leads to impaired responsiveness to IL-2 in the face of limited IL-2 supply *in vivo* [70]. Therefore, the surface CD25 expression on a per cell basis may also have functional importance and contribute to the enhanced suppression seen by Treg cells cultured in the presence of IL-2/TGF-β1 and atRA compared with IL-2/TGF-β1 without atRA.

These results show that the expression of FOXP3 and CD25 are enhanced by atRA, which is in accordance with early studies from Sakaguchi *et al*. [30]. Later, Wu *et al*. [71] confirmed at a molecular level that FOXP3 controls the expression of CD25 on Treg cells. This is consistent with the whole genome study by Saldon *et al*. [72] showing that CD25 is a target gene of FOXP3.

Battaglia *et al*. [73] showed for the first time a direct effect of RAPA on Treg cells, also suggesting that RAPA may be a useful adjunct to induce Treg cells in vitro [54]. Since then, a large amount of information has accumulated, documenting the promoting effect of RAPA on the expansion [34–36,38,74] and the induction of human Treg cells [13,39,55,60,75–82]. Nevertheless, only a few studies have addressed the effects of RAPA in combination with atRA on the induction of Treg in short-term cultures.

Our culture conditions, lasting 5 days, showed that RAPA alone and in combination with atRA did not enhance the expression of FOXP3, and therefore did not increase the number and proportion of Treg.

These results are apparently in contrast with the widely accepted Treg-promoting effect of RAPA, and highlight the complexities of mTOR inhibition [83]. Short-term exposure to RAPA inhibits mTORC1 complex but enhances the mTORC2 signal, while prolonged exposure to RAPA is necessary to inhibit both signals [84]. Zeng *et al*. showed that the genetic deletion of RAPTOR, a modification that interrupts the mTORC1 signal only, inhibits Treg, causing autoimmune disease [85]. Furthermore, this phenotype may be rescued by the simultaneous inhibition of mTORC2. Additionally, Delgoffe *et al*. [86] showed that the generation of Treg requires the simultaneous inhibition of mTORC1 and mTORC2. Therefore, it is not surprising that our experimental conditions, with brief exposure to RAPA, did not promote the induction of Treg. Our results are also compatible with results from Hippen *et al*. [13] and Qian *et al*. [77] showing a Treg promoting effect after 14 days of culture.

The concentration of RAPA may also be a critical factor in the induction and expansion of Treg cells. Levitzky *et al*. [87] evaluated the generation of Treg cells in alloimmune responses utilizing a mixed lymphocyte reaction, and showed that RAPA augmented the production of new Treg cells at low (sub-therapeutic) concentrations, but inhibited their generation at higher (5–10 ng/ml, therapeutic) concentrations. In our experimental conditions, Treg cells were induced with RAPA at 100 nM, equivalent to 91.41 ng/mL.

Independently, as shown in the functional experiments, Treg generated in the presence of RAPA were more suppressive than other tested conditions. The results shown are in accordance with Valmori *et al*. [75], who showed that RAPA has an inhibitory effect on the growth of human Treg as well as non-Treg cells and that this effect is related to enhanced suppressive function.

To exert their function, Treg cells similarly as conventional T cells need to enter peripheral tissues, which depend on the expression of specific homing molecules [88]. Thus therapeutic approaches using Treg cells must take into account proper tissue homing. Recent evidence indicates that activated Treg cells can be found as residents in many tissues, implying that these cells not only express specific tissue tropism but that they have a heterogeneous homing potential [89]. For example, human Treg cells isolated from cord blood express the gut-homing integrin α4β7, while the majority of adult nTreg show lower expression of this receptor and an increased expression of CCR4, implying that changes in homing receptor may allow Treg cells to leave secondary lymphoid organs and enter the different peripheral tissues. Conversely, Tomura et al. reported that during an inflammatory response, tissue resident Treg cells migrate to local lymph nodes where they may suppress immune responses through a direct action on antigen-presenting cells [90]. Recent reports indicate that a large proportion of naïve human peripheral blood Treg cells express CD62L and CCR7, a combination of receptors that should allow Treg cells to enter secondary lymphoid tissues [91], while effector/memory Treg cells express new adhesion molecules such as ligands for E and P selectins, ICAM-1, β1integrin, LAF-1 as well as chemokine receptors CCR2, CXCR3 and CCR6, implying that after Treg cells priming in secondary lymphoid organs they may acquire tropism to different inflamed tissue [92]. Thus, current information points to the notion that Treg cells exert their suppressive activity both at the draining lymph nodes as well as in the periphery. Activation of Treg cells at the T cell zone of lymphoid organs leads to the loss of CCR7, a decrease in CD62 expression with an increase in the expression of other homing receptors, supporting homing of Treg cells to the peripheral inflamed tissue.

Therefore, we were interested in the expression of activation and homing molecules on the obtained cells and its modulation under the different culture conditions. CD45RA is distinctive of immature, non-activated lymphocytes. We observed that Tn cells decrease the expression of CD45RA during the culture, whereas culture in the presence of atRA showed the strongest reduction.

Miyara *et al*. [93] have suggested that peripheral human FOXP3+CD4+ T cells may be classified into three subsets: CD45RA+FOXP3lo resting or naïve Treg cells, CD45RA-FOXP3hi activated or effector Treg cells, and CD45RA-FOXP3lo non-Treg cells. The first two have shown to be suppressive *in vitro* while the last phenotype secretes cytokines and is non-suppressive. The phenotype of the Treg cells obtained in our settings shows only one FOXP3-positive population, and it is not possible to distinguish between FOXP3hi and FOXP3lo population. In our experimental setting, all culture conditions expressed low levels of CD45RA on the Treg cells in comparison with the starting Tn cell population. Following the proposal by Miyara *et al*., [93] these cells would correspond to activated Treg cells. Comparing our different culture conditions, we observed that atRA induced a greater proportion of CD45RA-negative cells, which would correspond to the described activated phenotype. Treg cells generated in the presence of RAPA showed higher CD45 RA expression than Treg cells cultured in the presence of atRA, suggesting that RAPA cultured cells have a more resting or naïve phenotype in comparison with atRA cultured cells. Miyara *et al*. [93] showed that while CD45RA-FOXP3+ cells (activated Treg cells) display suppressive activity, they rapidly die. On the contrary, CD45RA+FOXP3low cells (resting Treg cells), proliferated and converted into activated Treg cells *in vitro* and *in vivo*. Arroyo Hornero *et al*. [94] showed recently in expanded human Treg cells a similar suppressive capacity of CD45RA+ and CD45RA- Treg cells. A similar suppressive activity has been recently confirmed by Canavan JB et al. [95] showing an enhanced stability of expanded CD45RA+cells *in vitro*. The diminished expression of CD45RA+ by atRA may also be in accordance with the enhanced proliferation of memory T cells by atRA as suggested by Hill *et al*. [22]

The expression of CD62L is characteristic of immature T cells and central memory cells, allowing lymphocytes to enter secondary lymphoid tissues. We observed that the presence of atRA generated a cell population with a reduced expression of CD62L in comparison with IL-2/TGF-β1 alone, which parallels the expression of CD45RA and is consistent with an activated phenotype. The addition of RAPA showed no difference in comparison with IL-2/TGF-β1 alone on the expression of CD62L. These results could serve as a complement to reports that indicate that CD62L-positive Treg cells protect against lethal acute graft versus host disease in a rodent model [96].

From a therapeutic standpoint, it would be optimal for Treg cells to accomplish their action in certain pre-defined organs without inducing general immunosuppression. Issa *et al*. [97] confirmed in a series of xenograft experiments the hypothesis that different populations of Treg cells display distinct efficacy *in vivo* based on their expression of tissue-specific homing molecules. Additionally, Engelhardt *et al*. [98] showed in allogeneic stem cell transplanted patients that increased frequency of Treg cells expressing skin-homing molecules or gut-homing molecules is associated with reduced risk of skin or gut acute graft versus host disease, respectively. Accordingly, it would be advantageous if Treg cells express homing molecules that allow their enrichment in certain target organs, depending on the clinical context.

Treg cells manufactured with certain homing properties represent a suitable therapy with potential applications in inflammatory bowel disease [99–101], allergy to food antigens [102], and inducing tolerance in bone marrow transplantation as well as solid organ transplantation [102,103]. Homing molecules to the intestine [104] and bone marrow [105] have been well characterized. Compelling evidence suggests that similarly as conventional naïve T cells, the migratory properties of FOXP3+ regulatory T cells are influenced by organ-specific dendritic cells and the tissue microenvironment [106]. Recent evidence from *in vitro* culture systems showed that, in the presence of atRA, dendritic cells from mesenteric lymph nodes induce Treg homing to mucosal tissues, while dendritic cells from peripheral LN polarized Treg cells to migrate to skin [88]. These results demonstrated that Treg cells could acquire organ-specific homing in response to specific environmental signals. In consequence, the expression of these molecules on the induced cells was analyzed, including Treg as well as non-Treg cells.

β7, a subunit of α4β7 integrin is a ligand of MAd-CAM-1, while CCR9 is a ligand of CCL25. Both molecules allow the migration of cells toward the lamina propria of the small intestine. CXCR-4, also known as fusin or CD184 is a ligand for Stromal Derived Factor-1 (SDF-1) a chemotactic molecule that allows the migration of lymphocytes to the bone marrow.

We observed that atRA induced β7 in all CD4+ cells, including FOXP3-positive and -negative cells, although the expression was higher on Treg cells (Fig 3B).

Kang *et al. [107]* used human and mouse Treg cells to demonstrate that retinoids generate a set of Treg cells with lasting suppressive activity and special tropism to the gut and associated tissue. They found that conventional FoxP3+ T cells express not only α4β7 and CCR9 but also other trafficking receptors such as CD103, E-selectin, P-selectin, CCR7, CCR9 and CXCR5. However, Treg cells induced in the presence of atRA and TGF-β, uniquely express the gut homing receptors α4β7 and CCR9 and do not express the other studied homing markers, except for mouse induced Treg cells, that also express CD103. Functional experiments also confirmed that atRA induced Treg cells preferentially migrate to the small intestine lamina propria in vivo.

More recently Duhen *et al*. [108] reported on the identification and characterization of phenotypically and functionally different human Treg populations expressing various combinations of adhesion and chemokine receptors. They argue this may enable Treg cell migration to specific tissues and underline the importance of defining the mechanisms by which Treg cells acquire transcription factors and specific homing receptors. The results shown here on the role of atRA on the acquisition of the gut-homing receptors may help to shed some light on this phenomenon.

In the setting of these experiments, CCR9 showed inconsistent results with large inter-individual disparities. Ohoka *et al*. [109] reported that while TCR and CD28 stimulation induce NFATc1 and NFATc2 translocation to the nucleus, prolonged TCR stimulation for more than 24 hours induces retention inside the nucleus of the isoform NFATc1. They also showed that NFATc1 inhibits binding of the complex retinoic acid receptor/retinoid X receptor (RAR/RXR) to a critical retinoic acid response element in the CCR9 promoter region. Therefore, the inconsistent expression of CCR9 in the presence of atRA may be related to the prolonged TCR stimulation in our experimental setting. Jhunjhunwala *et al*.[37] evaluated the expression of CCR9 in atRA and RAPA induced murine Treg cells showing that atRA up-regulates and RAPA does not modify its expression, while atRA with RAPA gives rise to three distinct Treg cells populations based on the expression of CCR9 and CD103. Chen LC *et al*. [110] have recently studied CCR9 expression in expanded murine Treg cells showing that only 50% of Treg up-regulate CCR9 when stimulated by atRA. Additionally, they showed that CCR9 and α4β7 do not follow the same signal pathways, whereas the expression of CCR9 is mTORC1-dependent. The complexity of CCR9 expression is best highlighted by the recognition by Evans-Marin *et al*. [111] that CCR9 is not only a gut homing molecule but also a molecule that regulates the development of Treg cells. Retinoic acid-mediated expression of α4β7 and CCR9 does not respond to identical mechanisms. While retinoic acid receptors and retinoid X receptors are both essential for optimal CCR9 expression, only retinoic acid receptors are key for α4β7 expression [112]. Further studies are necessary to fully clarify the mechanisms and role of CCR9 expression on Treg cells.

The interpretation of intestinal homing experiments in graft versus host disease under diminished and increased vitamin A signaling is hampered by the fact that retinoic acid signaling affects both effector and regulatory cells [113,114].

Regarding the potential clinical applications of induced Treg cells, their efficacy to treat inflammatory bowel disease may be hampered by a variable inter-individual expression of CCR9. However, Treg cells therapy represents a new potential treatment to manage inflammatory bowel disease or to prevent and treat rejection in intestinal transplantation [6].

The expression of CXCR4, the ligand of stromal-derived-factor-1 (SDF-1), which is constitutively expressed in the bone marrow, was also examined. It has been reported that CXCR4 in smooth muscle cells is up-regulated by TGF-β/Smad3 [115]. We found that atRA, in addition to IL-2/TGF-β1, did not induce any further increase in the expression of CXCR4, while RAPA enhanced its expression. atRA plus RAPA induced the highest expression of CXCR4. These effects were observed on Treg cells as well as on CD4+ non-Treg cells (Fig 3B). This fact highlights the possibility of manufacturing Treg cells with bone marrow homing properties.

In contrast to recognized knowledge, it has been reported that naïve, non-differentiated CD4+ T cells may also reach peripheral tissues such as lung and liver, even in absence of inflammation [116]. Expression of CD62L and CCR7 on naïve T cells are crucial to the migration of these cells into secondary lymphoid organs while the expression of tissue-specific homing receptor such as α4β7 and CCR9 and CLA target effector and Treg cells to the gut and skin respectively. However, recent reports indicate that human naïve CD4 T cells express discreet amounts of integrin α4β7 [117], while in the mouse recent thymic emigrants are known to express CCR9, an indication that these molecules may participate in naïve T cell entry to peripheral tissue. It has been reported that CXCR4 is also expressed at higher levels in naïve CD4+ T cells than in memory T cells[118]. Although the ultimate mechanism involved in the entrance of naïve T cells into the parenchyma is unknown, the expression of low levels of these tissue homing receptors on naïve T cells plus the expression of chemokine receptor CCR7 provides some clues as to the mechanism involved. The presence of naïve T cells in non-lymphoid organs opens the possibility that these cells may participate in preventing local infection as a result of tissue injury. Another more interesting possibility is that these cells may participate in the acquisition of tolerance to tissue antigens, perhaps through the induction of local Treg cells.

Collectively, our results are in accordance with the results obtained in induced murine Treg cells by Jhunjhunwala *et al*. [37] and in expanded human Treg cells by Scotta *et al*. [38], suggesting that Treg cells might be customized to confer different migratory capacities.

Based on the mentioned literature a suggested characterization of the induced Treg cells, depending on the expression of the studied surface markers, could be attempted. Treg cells cultured in IL-2/TGF-β1 could be characterized as having a non-activated phenotype (CD45RA+), with the ability to enter lymph nodes (CD62L+), and a low homing capacity to bone marrow (CXCR4 low) and the intestine (CCR9 low, β7 low). Treg cells cultured in IL-2/TGF-β1 and atRA could be characterized as having a higher proportion of activated Treg cells (higher proportion of CD45RA-), with a low capacity to enter the lymph nodes (low CD62L), yet endowed with the capability to home to the intestine (β7 high), especially to the lamina propria. Treg cells induced in the presence of IL-2/TGF-β1 and RAPA could be characterized as non-activated or resting Treg cells, possibly endowed with bone marrow homing capacity. Treg cells induced in the presence of IL-2/TGF-β1, atRA, and RAPA have a non-activated phenotype with a homing potential to both the bone marrow and the intestine.

Comparing the effects of the different culture conditions on Treg cells and non-Treg cells (Fig 3B), we observed the same expression pattern of CD45RA, CD62L, CXCR4, CCR9 and β7. These results are compatible with Duhen *et al*.[108], who identified distinct subpopulations of Treg cells in human blood expected to co-localize with different helper T cells. Our findings in conjunction with Duhen *et al*. suggest that the mechanisms by which homing molecules are induced are similar in regulatory and effector cells.

The experiments shown are consistent with the possibility of inducing customized Treg cells, depending on the specific pathology to be treated. Functional homing experiments should demonstrate if the patterns observed in these experiments could be applied to the clinic.

The expression of FOXP3, a gene considered a crucial factor on Treg cells identity and function is considered mandatory on Treg cells [51]. The FOXP3 expression on thymic Treg cells has shown to be stable [47,119], whereas its expression on induced Treg cells has proven to be unstable[120]. To assess the stability of the generated Treg cells in the described culture conditions, the methylation of the CNS2 region of the *FOXP3* gene was studied. Previous studies have shown that the stable expression of FOXP3 is regulated at the epigenetic level [121]. It has also been demonstrated that the CNS2 region in TGF-β1 induced Treg cells is highly methylated [122]. Our study performed in 11 CpGs in the CNS2 showed that TGF-β1, IL-2, atRA and RAPA have no DNA demethylating effect under the conditions of the present study. These results are in accordance with the experiments performed by Lu *et al*. [50,123], and by Xu *et al*. [124], showing that atRA promotes the development and maintenance of TGF-β1 induced Treg cells via histone modification, but not through DNA demethylation. Schmidt *et al. [39]* reported first that atRA and RAPA have no demethylating effect on induced human Treg cells. Our results confirmed these results with some methodological differences; in our case, utilizing pyrosequencing focused on the CNS2. Additionally, we demonstrated that RAPA without atRA did not modify the methylation of CNS2 on induced human Treg. The elevated CNS2 methylation justifies the capacity of induced FOXP3 cells to suppress effector cells in short-term *in vitro* cultures, but also their inability to sustain FOXP3 expression and regulate the immune response *in vivo*, as shown by Schmidt *et al*. [39].

In conclusion, the elevated CNS2 methylation on induced Treg remains a critical hurdle on the path to obtaining clinically useful induced Treg cells. Other ongoing approaches should be considered to overcome the elevated CNS2 methylation. Ten-eleven translocation enzymes (TET) catalyze the oxidation of 5-methylcytosine, actively decreasing DNA methylation. Yue *et al*. [125] have shown that TET proteins mediate the demethylation of CNS1 and CNS2 in the thymus and that TET-deficient mice have markedly unstable FoxP3 expression. After demonstrating that vitamin C increases TET activity, the authors went on to show that vitamin C contributes to demethylate and stabilize FoxP3 *in vitro* and *in vivo*, in rodents as well as in humans. Moreover, the authors showed that induced Treg cells conditioned with vitamin C potentiate the suppressor function of human induced Treg cells to the levels observed in Treg cells isolated and expanded directly from human peripheral blood. Consistent with the previous research, Sasisharan Nair *et al*.[126] demonstrated that vitamin C is required for the CNS2 demethylation mediated by TET proteins, which is essential for FoxP3 expression.

Recently, Miyara *et al*. [127] showed that a combination of IL-2, RAPA, DNA methyltransferase and histone deacetylase inhibitors are able to enhance the expression of Foxp3 on Treg cells expanded *in vitro*, and control a murine xenogeneic graft versus host reaction *in vivo*. It remains to be seen whether a similar combination of factors may contribute to demethylate and stabilize induced Treg cells. Very recently, Okada M *et al*. [128] described a novel approach utilizing clustered regularly interspaced short palindromic repeats (CRISPR)-dCas9 based technology to target the catalytic domain of p300 histone acetyltransferase, with guide RNAs targeted to the *Foxp3* promoter locus. The authors showed that mouse primary T cells increased the acetylation of the promoter locus, and strongly activated and stabilized Foxp3 expression, particularly with TGF-β, even under inflammatory conditions. Validation of the efficiency of this approach is still in progress; however, the specificity of this strategy may represent a major development to advance induced Treg cells into an applied cell therapy.

Our functional studies compared the suppressive activity of Treg induced under different conditions. Suppression assays *in vitro* are complex, and may reflect the situation *in vivo* only with limitations. However considering these limitations, it may allow conclusions particularly when comparing cells induced under different conditions. Selecting viable Treg cells based on the expression of FOXP3 is not possible due to the permeabilization. The option of selecting the entire cell population after culture would include an important proportion of other cell linages. Therefore, our setting involved labeling the CD25+ and CD127- population, followed by cell sorting, which allowed for the isolation a highly enriched Treg population (98.3% see Fig 5C) suitable for the suppression assays. Additionally, we utilized non-labeled effector cells to control for the density of the cells in the suppression assay, which may hinder their access to activation stimuli, nutrition and cytokines [52].

The most effective suppression was observed with Treg cells obtained in the presence of RAPA, and the least effective with IL-2/TGF-β1 alone. The addition of atRA, although less efficient than RAPA, also had a major effect on the suppressive capacity evaluated *in vitro*. As shown, Treg cells induced with atRA have a higher CD25 expression, and this difference may account for the enhanced response, as discussed. Schmidt et al. [39] studied the effects of induced Treg on CD4+ and CD8+ responder cells, showing that human IL-2/TGF-β1 induced Treg cells cultured in the presence of RAPA with atRA have enhanced suppressive effect as compared to with atRA alone, which is in accordance with our results. We also evaluated RAPA in comparison to atRA, showing that atRA Treg cells induced without RAPA have significantly lower suppressive activity compared to Treg cells generated with RAPA. On the other hand, in two donors analyzed, Treg cells generated with IL-2/TGF-β1 either in the absence or in the presence of RAPA showed a comparable range of FOXP3 expression. This suggests that the higher suppressive activity of Treg cells generated in the presence of RAPA may not be explained solely by their higher expression of FOXP3. Of note, Treg cells induced in the presence of RAPA with atRA had a lower proportion of CD45RA+ cells in comparison to Treg cells cultured in the presence of RAPA without atRA. As explained below CD45RA+ cells may be more suppressive explaining at least partially the observed differences. Sorting of the Treg cells for the suppression assay was based on the enrichment of CD4+CD25highCD127- cells. In cells sorted from two different donors, we observed that the expression of FOXP3 was not uniform in the different cultures, being lowest in the atRA and highest in RAPA cultured cells (S5 Fig). These results may suggest that the absence of CD127 expression, particularly on atRA induced Treg cells may not accurately correlate with the expression of FOXP3 as shown by others in human peripheral Treg cells [129]. Further experiments should disclose how the expression of CD127 correlates with the expression of FOXP3 in IL-2/TGF-β1, atRA and RAPA induced Treg cells.

It has been shown that a fine-tuned suppression of the mTOR axis is necessary for the function of Treg cells. Apostolidis *et al*. [130] have recently described a complex mechanism whereby Foxp3 increases the intracellular ceramide content, increases the activity of the phosphatase PP2A and inhibits the mTORC1 complex, which translates into an increased inhibitory function of Treg cells. In accordance, Gerriets *et al*. [131] recently showed that TLR mediated mTORC1 activation induces enhanced cell growth and proliferation but lower suppressive capacity. On the other hand, as inflammatory signals are reduced, Foxp3 can tilt the balance away from mTORC1 signaling, which translates into lower proliferation but higher suppressive capacity, and also correlates with glucose metabolism. Along these lines, our results with Treg cells induced in the presence of RAPA may reflect diminished mTORC1 signaling, which according to the mentioned study, is expected to translate into diminished proliferation but enhanced suppression.

The results shown in our study are also in accordance with Singh *et al*. [132], demonstrating that expanded Treg cells with RAPA have enhanced inhibitory capacity but not necessarily enhanced proliferation. Additionally, studies performed in human expanded Treg cells show that the functional differences in RAPA-conditioned Treg cells could be explained by RAPA-mediated inhibition of the cytokines IFN-g and IL-17 [15].

We observed that cultures in the presence of RAPA are related to higher CD45RA expression on Treg cells. Hoffmann *et al*. [133] showed that only CD45RA+ cells maintain the expression of CD62L and CCR7, as well as the phenotypic and functional characteristics of Treg cells after expansion. Therefore, CD45RA- cells may include cells which are functionally distinct from Treg cells. This result is in accordance with Golovina *et al*. [66], and Miyara *et al*., [93] showing that the CD45RA+Treg cells population in expanded Treg cells, also called the naïve population, has a higher suppressor activity than CD45RA- cells. Our results are in line with the mentioned studies confirming that Treg cells induced with RAPA or a combination of RAPA and atRA display robust suppressive function across studies, despite differences in the experimental conditions. The expression of CD62L may also be a determining factor with regards to the suppressive function of induced Treg cells. Experiments by Ermann *et al*. [96] and Huter *et al*. [134] highlight the importance of CD62L expression, which is a determining factor of entry to the lymph nodes. Our results show similar CD62L expression by IL-2/TGF-β1 and IL-2/TGF-β1+RAPA cultured cells, although we observed enhanced suppression by IL-2/TGF-β1+RAPA cultured cells in the *in vitro* suppression assay.

In conclusion, the findings of this study corroborate previous studies indicating that atRA effectively contributes to the generation of human Treg cells and that the optimal concentration of atRA is 100 nM. This study also shows that, while atRA promotes the expression of β7, RAPA enhances the expression of CXCR4, which could create the opportunity to direct Treg cells to the intestine or the bone marrow. Treg cells generated in the presence of RAPA showed the most potent inhibition in the *in vitro* suppression assay. The high methylation rate of the critical *FOXP3* gene in these Treg cells underscores the difficulties to be addressed in the future in order to produce a stable Treg cell lineage.

## Supporting information

S1 Fig Gating strategy, representative data and differential FOXP3 and CD25 expression analysis for atRA induced Treg cells. Representative gating strategy utilized for the characterization of CD4+CD25+FOXP3+ Treg cells. Shown cells were cultured for 5 days with IL-2/TGF-β1/ATRA 100 nM. Autofluorescence parameters are shown in the upper row. Forward and side-scatter, doublets exclusion, gating on live cells, gating on live CD4+ T cells, FOXP3 isotype and CD25+FOXP3+ on the live CD4 T cells gate is shown in the lower row. (B) Representative dot plots of the in Fig 1 shown CD4+ T cells obtained under different conditions. (C) The left upper scheme shows the quadrants analyzed in C1 and C2 and the right upper scheme shows the quadrants analyzed in C3 and C4. Cells were gated on live CD4+ cells. (C1) Comparison of FOXP3- and FOXP3+ cells percentage in the CD4+CD25+ quadrants. (C2) Comparison of the CD25 nMFI in FOXP3- and FOXP3+ cells in the CD4+CD25+ quadrants. (C3) Comparison of CD25- and CD25+ cells percentage in the CD4+FOXP3+ quadrants. (C4) Comparison of the FOXP3 nMFI in CD25- and CD25+ cells in the CD4+FOXP3+ quadrants. Significance was calculated by two-way repeated-measures ANOVA and Sidak's post-test. *p < 0.05; **p < 0.01; ***p < 0.001; ****p < 0.0001 for n = 7–8 donors in independent experiments.
(TIF)

S2 Fig Representative data and differential FOXP3 and CD25 expression analysis for RAPA induced Treg cells. (A) Representative dot plots of the CD4+ T cells obtained after culturing under different conditions for 5 days as shown in Fig 2. B The left upper scheme show the quadrants analyzed in Fig B1 and B2 and the right upper scheme show the quadrants analyzed in Fig B3 and B4. Cells were gated on live CD4+ cells. (B1) Comparison of FOXP3- and FOXP3+ cells percentage in the CD4+CD25+ quadrants. (B2) Comparison of the CD25 nMFI in FOXP3- and FOXP3+ cells in the CD4+CD25+ quadrants. (B3) Comparison of CD25- and CD25+ cells percentage in the CD4+FOXP3+ quadrants. (B4) Comparison of the FOXP3 nMFI in CD25- and CD25+ cells in the CD4+FOXP3+ quadrants. Significance was calculated by two-way repeated-measures ANOVA and Sidak's post-test. *p < 0.05; **p < 0.01; ***p < 0.001; ****p < 0.0001 for n = 7–8 donors in independent experiments.
(TIF)

S3 Fig Gating strategy and representative data for homing markers expression by induced Treg cells. Representative gating strategy utilized for the analysis of homing markers expression on cells cultured for 5 days with IL-2/TGF-β1/ATRA 100 nM and RAPA 100 nM. Forward and side-scatter, doublets exclusion, gating on live CD4+ T cells, FOXP3 isotype and CD25+FOXP3+ on the live CD4 T cells was performed as in S1 Fig. Additionally, isotype controls for the homing molecules and PE-fluorescence minus one control (FMO) were performed. Non-Treg cells were defined as either CD25- or FOXP3- or both. (A) and (B) show representative figures of the gating strategy for two different culture conditions and homing markers, as shown. (C) Representative dot plots of the expression of homing molecules by CD4+ live cells cultured in the presence of IL-2 (100U/mL), TGF-β1 (5ng/mL), atRA (100nM) and RAPA (100nM) as shown.
(TIF)

S4 Fig Single methylation analysis of each CpG studied in the CNS2 region. (A) Scheme showing the 11 CpG sites analyzed within the first intron of human FOXP3 TDSR region (-2376 to -2263 from ATG, ENST00000376207). (B) Box and whisker plots showing the mean methylation values for each CpG shown as methylation percentage. n = 3–6 donors in independent experiments. Non-parametric Mann–Whitney U-test. *p < 0.05; **p < 0.01; ***p < 0.001; ****p < 0.0001.
(TIF)

S5 Fig Gating strategy utilized in the suppression assay. (A) Forward and side-scatter, doublets exclusion strategy as explained in the methods section and CD4+CD25+CD127- cells before sorting and after sorting. (B) Representative Dot Plots from 2 donors showing the expression of CD25 and FOXP3 in CD4+CD25highCD127- cells after sorting. (C) Comparison of the suppression capacity of different Treg:Teff ratios for each treatment. Two-way repeated measures ANOVA followed by Sidak’s Multiple-Comparison post-hoc test. (* = P<0.05, ** = P<0.01, *** = P<0.001, **** = P<0.0001). n = 4 independent experiments.
(TIF)

S1 Table Individual-level data behind results mentioned in the text and figures. The table shows individual data points for each donor, as mentioned in the manuscript text and figures, as indicated.
(XLSX)
