# Supplementary figures and images for: Single and combined effect of retinoic acid and rapamycin modulate the generation, activity and homing potential of induced human regulatory T cells
Source: PLoS One. 2017 Jul 26;12(7):e0182009. doi: 10.1371/journal.pone.0182009 (PMC5529012; doi:10.1371/journal.pone.0182009)

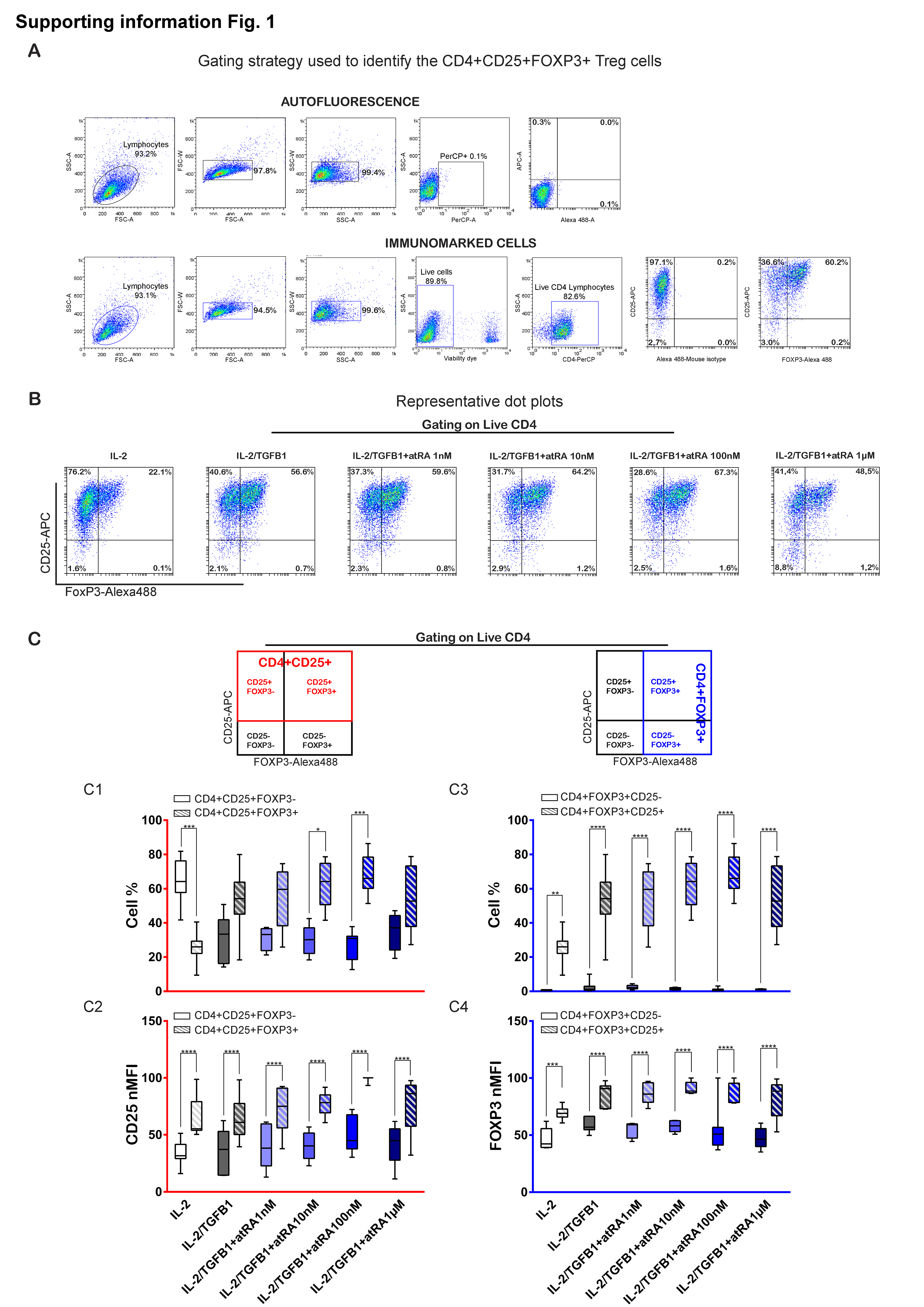

Supplement: S1 Fig — Representative gating strategy utilized for the characterization of CD4+CD25+FOXP3+ Treg cells. Shown cells were cultured for 5 days with IL-2/TGF-β1/ATRA 100 nM. Autofluorescence parameters are shown in the upper row. Forward and side-scatter, doublets exclusion, gating on live cells, gating on live CD4+ T cells, FOXP3 isotype and CD25+FOXP3+ on the live CD4 T cells gate is shown in the lower row. (B) Representative dot plots of the in Fig 1 shown CD4+ T cells obtained under different conditions. (C) The left upper scheme shows the quadrants analyzed in C1 and C2 and the right upper scheme shows the quadrants analyzed in C3 and C4. Cells were gated on live CD4+ cells. (C1) Comparison of FOXP3- and FOXP3+ cells percentage in the CD4+CD25+ quadrants. (C2) Comparison of the CD25 nMFI in FOXP3- and FOXP3+ cells in the CD4+CD25+ quadrants. (C3) Comparison of CD25- and CD25+ cells percentage in the CD4+FOXP3+ quadrants. (C4) Comparison of the FOXP3 nMFI in CD25- and CD25+ cells in the CD4+FOXP3+ quadrants. Significance was calculated by two-way repeated-measures ANOVA and Sidak's post-test. *p < 0.05; **p < 0.01; ***p < 0.001; ****p < 0.0001 for n = 7–8 donors in independent experiments. (TIF) [file pone.0182009.s001.tif]

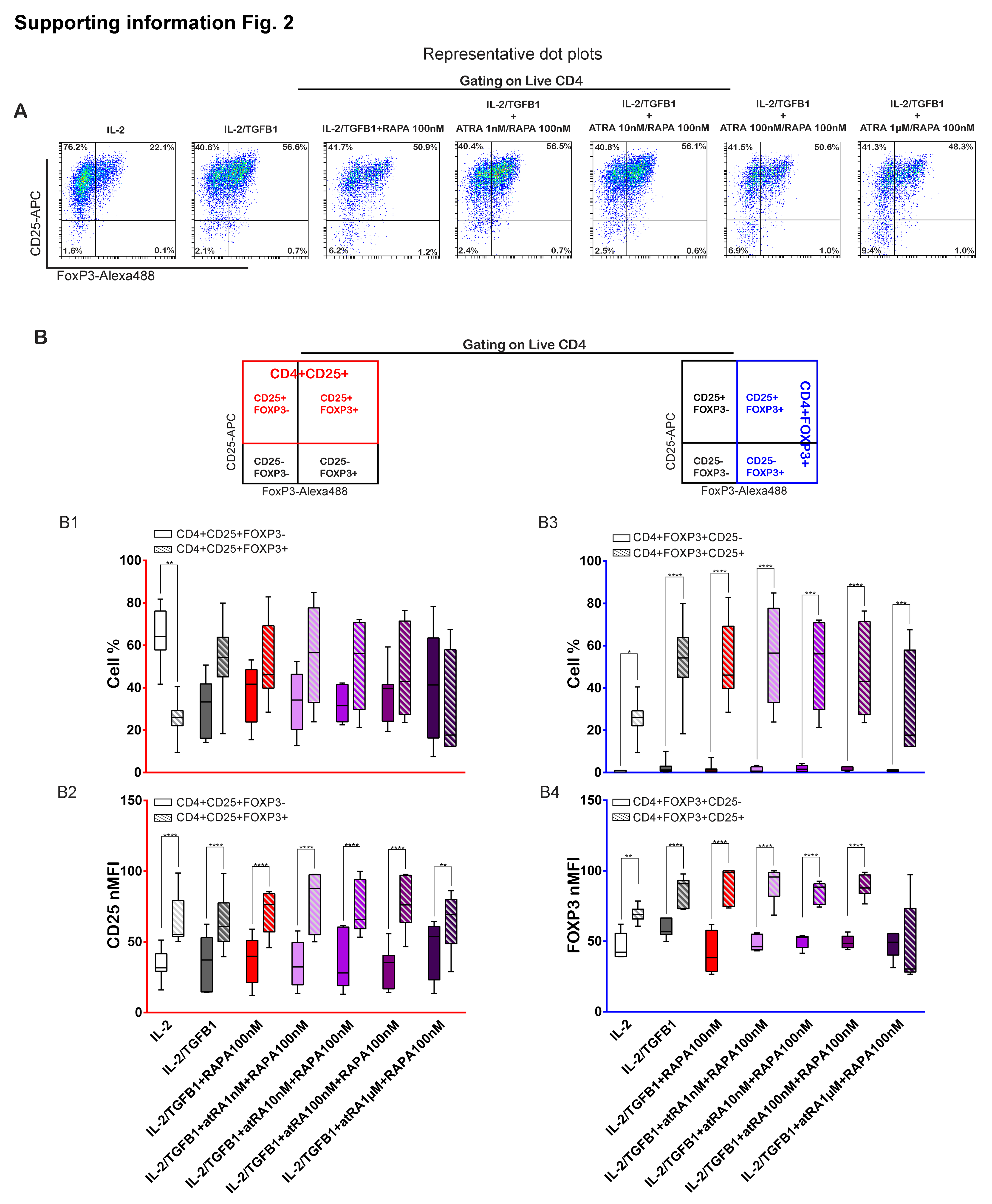

Supplement: S2 Fig — (A) Representative dot plots of the CD4+ T cells obtained after culturing under different conditions for 5 days as shown in Fig 2. B The left upper scheme show the quadrants analyzed in Fig B1 and B2 and the right upper scheme show the quadrants analyzed in Fig B3 and B4. Cells were gated on live CD4+ cells. (B1) Comparison of FOXP3- and FOXP3+ cells percentage in the CD4+CD25+ quadrants. (B2) Comparison of the CD25 nMFI in FOXP3- and FOXP3+ cells in the CD4+CD25+ quadrants. (B3) Comparison of CD25- and CD25+ cells percentage in the CD4+FOXP3+ quadrants. (B4) Comparison of the FOXP3 nMFI in CD25- and CD25+ cells in the CD4+FOXP3+ quadrants. Significance was calculated by two-way repeated-measures ANOVA and Sidak's post-test. *p < 0.05; **p < 0.01; ***p < 0.001; ****p < 0.0001 for n = 7–8 donors in independent experiments. (TIF) [file pone.0182009.s002.tif]

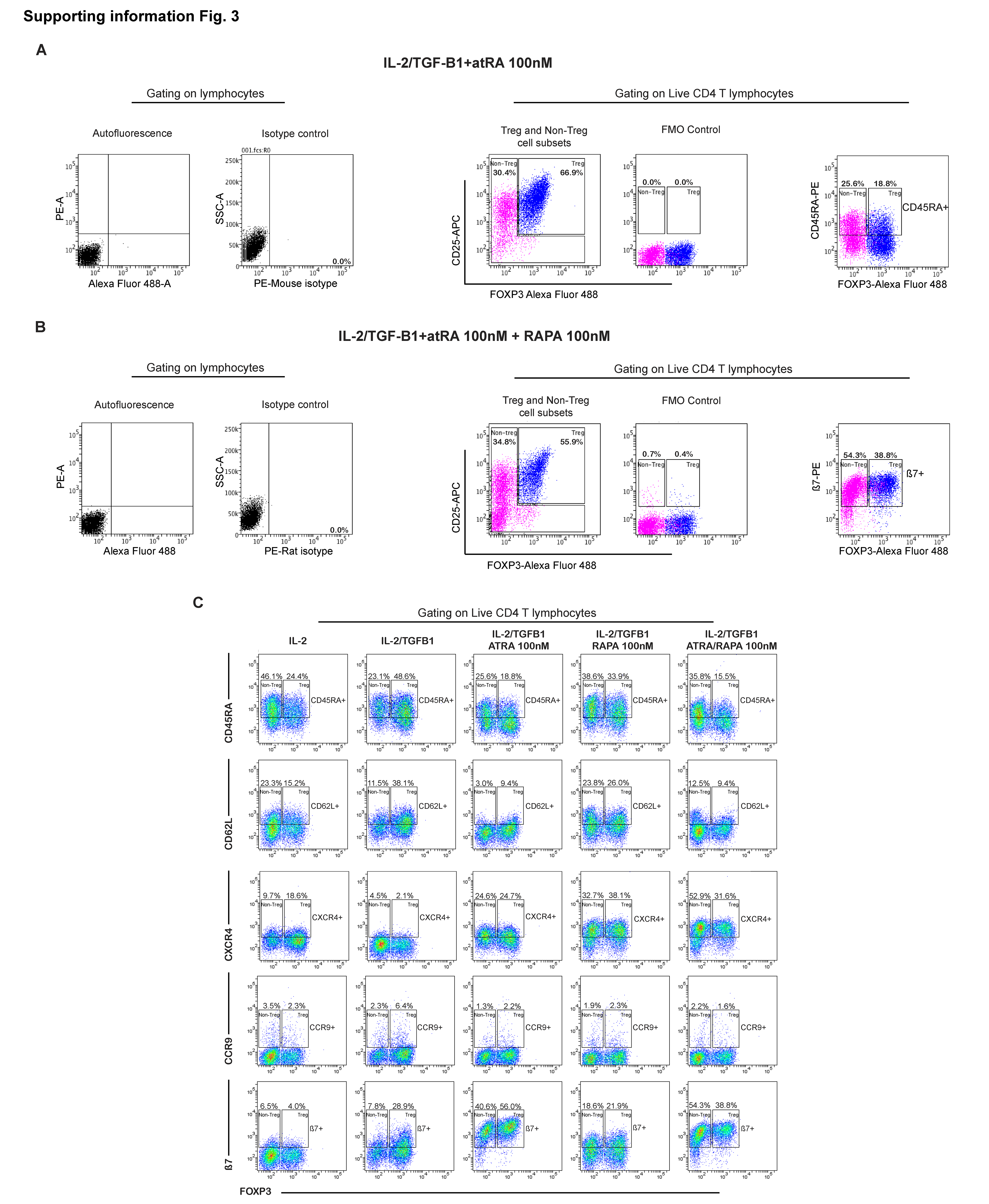

Supplement: S3 Fig — Representative gating strategy utilized for the analysis of homing markers expression on cells cultured for 5 days with IL-2/TGF-β1/ATRA 100 nM and RAPA 100 nM. Forward and side-scatter, doublets exclusion, gating on live CD4+ T cells, FOXP3 isotype and CD25+FOXP3+ on the live CD4 T cells was performed as in S1 Fig. Additionally, isotype controls for the homing molecules and PE-fluorescence minus one control (FMO) were performed. Non-Treg cells were defined as either CD25- or FOXP3- or both. (A) and (B) show representative figures of the gating strategy for two different culture conditions and homing markers, as shown. (C) Representative dot plots of the expression of homing molecules by CD4+ live cells cultured in the presence of IL-2 (100U/mL), TGF-β1 (5ng/mL), atRA (100nM) and RAPA (100nM) as shown. (TIF) [file pone.0182009.s003.tif]

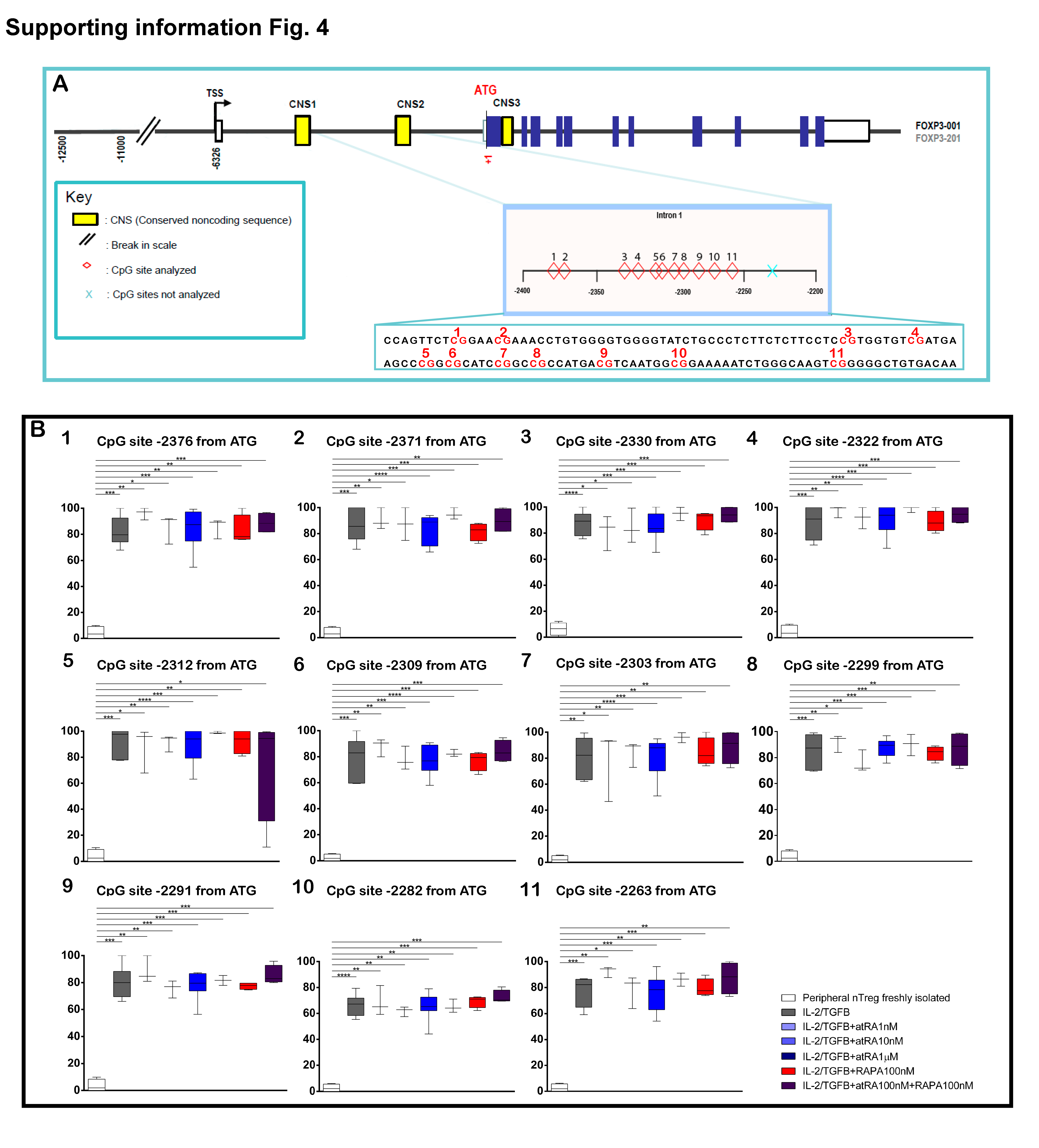

Supplement: S4 Fig — (A) Scheme showing the 11 CpG sites analyzed within the first intron of human FOXP3 TDSR region (-2376 to -2263 from ATG, ENST00000376207). (B) Box and whisker plots showing the mean methylation values for each CpG shown as methylation percentage. n = 3–6 donors in independent experiments. Non-parametric Mann–Whitney U-test. *p < 0.05; **p < 0.01; ***p < 0.001; ****p < 0.0001. (TIF) [file pone.0182009.s004.tif]

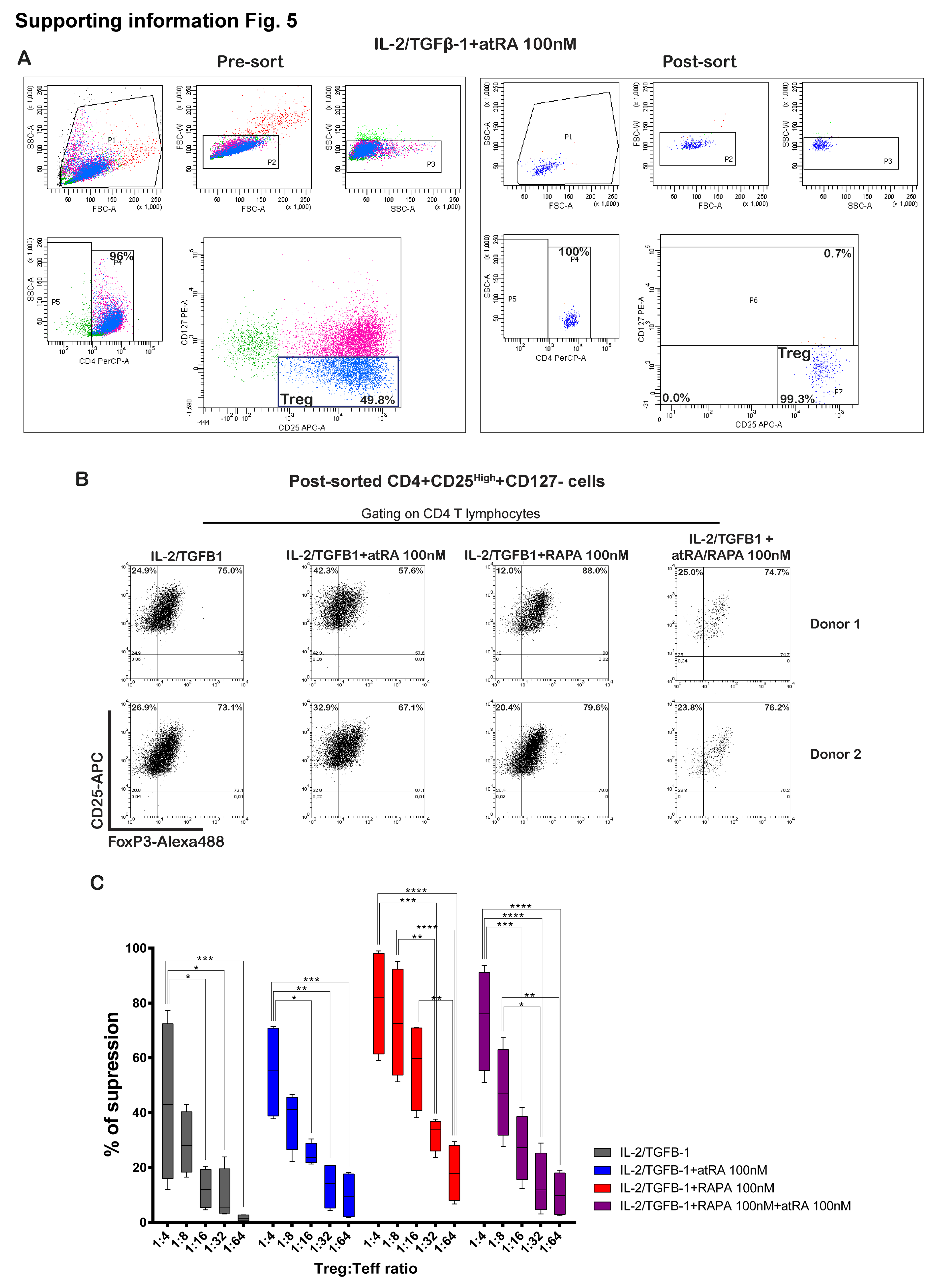

Supplement: S5 Fig — (A) Forward and side-scatter, doublets exclusion strategy as explained in the methods section and CD4+CD25+CD127- cells before sorting and after sorting. (B) Representative Dot Plots from 2 donors showing the expression of CD25 and FOXP3 in CD4+CD25highCD127- cells after sorting. (C) Comparison of the suppression capacity of different Treg:Teff ratios for each treatment. Two-way repeated measures ANOVA followed by Sidak’s Multiple-Comparison post-hoc test. (* = P<0.05, ** = P<0.01, *** = P<0.001, **** = P<0.0001). n = 4 independent experiments. (TIF) [file pone.0182009.s005.tif]
